# Establishment and characterization of turtle liver organoids provides a potential model to decode their unique adaptations

**DOI:** 10.1038/s42003-024-05818-1

**Published:** 2024-02-22

**Authors:** Christopher Zdyrski, Vojtech Gabriel, Thea B. Gessler, Abigail Ralston, Itzel Sifuentes-Romero, Debosmita Kundu, Sydney Honold, Hannah Wickham, Nicholas E. Topping, Dipak Kumar Sahoo, Basanta Bista, Jeffrey Tamplin, Oscar Ospina, Pablo Piñeyro, Marco Arriaga, Jacob A. Galan, David K. Meyerholz, Karin Allenspach, Jonathan P. Mochel, Nicole Valenzuela

**Affiliations:** 1 SMART Pharmacology, Department of Biomedical Sciences, Iowa State University, Ames, IA USA; 2 3D Health Solutions Inc., Ames, IA USA; 3 Department of Ecology, Evolution, and Organismal Biology, Iowa State University, Ames, IA USA; 4 Department of Statistics, Iowa State University, Ames, IA USA; 5 Department of Veterinary Clinical Sciences, Iowa State University, Ames, IA USA; 6 Department of Biology, University of Northern Iowa, Cedar Falls, IA USA; 7 Department of Biostatistics and Bioinformatics, Moffitt Cancer Center, Tampa, FL USA; 8 Veterinary Diagnostic Laboratory, Iowa State University, Ames, IA USA; 9 Department of Human Genetics, University of Texas Rio Grande Valley, Brownsville, TX USA; 10 Department of Pathology, University of Iowa, Iowa City, IA USA; 11 Present Address: SMART Pharmacology, Precision One Health Initiative, University of Georgia, Athens, GA USA

**Keywords:** Adult stem cells, Transcriptomics

## Abstract

Painted turtles are remarkable for their freeze tolerance and supercooling ability along with their associated resilience to hypoxia/anoxia and oxidative stress, rendering them an ideal biomedical model for hypoxia-induced injuries (including strokes), tissue cooling during surgeries, and organ cryopreservation. Yet, such research is hindered by their seasonal reproduction and slow maturation. Here we developed and characterized adult stem cell-derived turtle liver organoids (3D self-assembled in vitro structures) from painted, snapping, and spiny softshell turtles spanning ~175My of evolution, with a subset cryopreserved. This development is, to the best of our knowledge, a first for this vertebrate Order, and complements the only other non-avian reptile organoids from snake venom glands. Preliminary characterization, including morphological, transcriptomic, and proteomic analyses, revealed organoids enriched in cholangiocytes. Deriving organoids from distant turtles and life stages demonstrates that our techniques are broadly applicable to chelonians, permitting the development of functional genomic tools currently lacking in herpetological research. Such platform could potentially support studies including genome-to-phenome mapping, gene function, genome architecture, and adaptive responses to climate change, with implications for ecological, evolutionary, and biomedical research.

## Abstract (web summary)

Multi-omic profiling on 3D liver organoids derived from multiple species may help foster future research on unique turtle adaptations, along with their evolutionary and biomedical implications.

## Introduction

Turtles are an ancient and enigmatic group of reptiles recognized for their distinctive morphology, longevity, tolerance to anoxia, and diverse sex-determining systems. Importantly, the study of turtles is both time-sensitive and critical, due to their at-risk status resulting from anthropogenic global environmental change, habitat loss, and overharvesting, among others^1,2^. Because of their biology and phylogenic position, turtles hold clues to unlock numerous biological mysteries, including current biomedical questions. Indeed, turtles are an emerging model for ecology, evolution, and human health^3^, currently studied to understand physiology, life histories, chromosome evolution, as ecotoxicology sentinels, and to decipher biological pathways for sexual development and reproduction^4–11^. But while reptile genomics is thriving, reptilian transgenics remains challenging despite pioneering in vivo gene editing in anole lizards^12^ and recent work in mourning geckos^13^. One reason is that turtle research is currently hindered due to the scarcity of in vitro tools, and additionally impeded by the slow maturation and seasonal reproduction of chelonians. Thus, developing methods that overcome these bottlenecks and increase their use in basic and applied research to study their remarkable adaptations is overdue. Stem cell-derived organoids^14^ are an attractive model for functional genomics as they form complex 3D structures that recapitulate the microanatomy and physiology of their tissue of origin^15,16^. Unlike conventional 2D cell cultures, adult stem cell-derived organoids have several advantages, including commonly being composed of multiple epithelial cell types also found in the tissue of origin, being able to be expanded continuously, and being able to self-renew and self-organize^17^.

Organoid technology has expanded recently from commonly used mice and human models to canines^18–23^ and multiple other vertebrates^24–26^. However, the only reptilian (*sensu lato*) organoids (i.e., excluding those derived from chicken intestines^27^), were generated from snake venom glands^28^, representing only a fraction of the vast Tree of Life. Expanding the taxonomic coverage of the 3D organoid technology will leverage unique adaptations that evolved in reptiles and other non-model species, broadening potential applications of this technology. Here we report the generation of liver organoids from painted turtles (*Chrysemys picta*), snapping turtles (*Chelydra serpentina*), and spiny softshell turtles (*Apalone spinifera*), and their characterization via histological staining, RNA-seq transcriptomics, single-nuclei RNA-seq, transmission electron microscopy, and mass spectrometry (MS)-based proteomics.

Painted turtles are well-adapted to overwintering conditions by supercooling their body and surviving in the ensuing hypoxic and ischemic conditions^29^. They are one of the most anoxia-tolerant tetrapods^30^, which, along with the slider turtle (*Trachemys scripta*), can survive for weeks without oxygen^31^. Because the liver is critical to the adaptive defense underlying their supercooling capacity and tolerance to anoxia^32,33^, the development of liver organoids may open the door for biomedical research related to these adaptations.

Indeed, certain human diseases cause hypoxia in vital organs, as reported in liver cirrhosis, an end-stage disease caused by chronic injuries to the hepatic tissue by drugs, alcohol, infections, and genetic disorders^34^. Acute injury to the liver can consequentially cause the upregulation of hypoxia-inducible factors (HIFs) to maintain homeostasis^34,35^. Furthermore, hepatic ischemia-reperfusion injury (IRI), a major clinical complication during liver transplantation, severe trauma, vascular surgery, and hemorrhagic shock^36^, seems improved by cold machine-perfusion of organs before transplantation^37^. Thus, understanding the capability of turtle liver organoids to survive hypoxia and anoxia may aid in discovering important proteins possibly implicated in future development of treatment options for IRI. For instance, antifreeze glycoproteins prolonged the survival of mouse intestinal organoids when incubated at 4 °C for up to 72 h^38^. Turtle liver organoids may help illuminate their unique adaptative strategies to overwintering which could benefit human organ preservation medicine. However, future repeatable and consistent functional assays will be necessary to utilize organoid technology to the fullest extent. Overall, the development of organoids from non-model species, such as turtles, can greatly impact biomedical research by exploiting a myriad of adaptations found across the Tree of Life.

## Results

### Growth and expansion of liver organoids

**Fig. 1:**
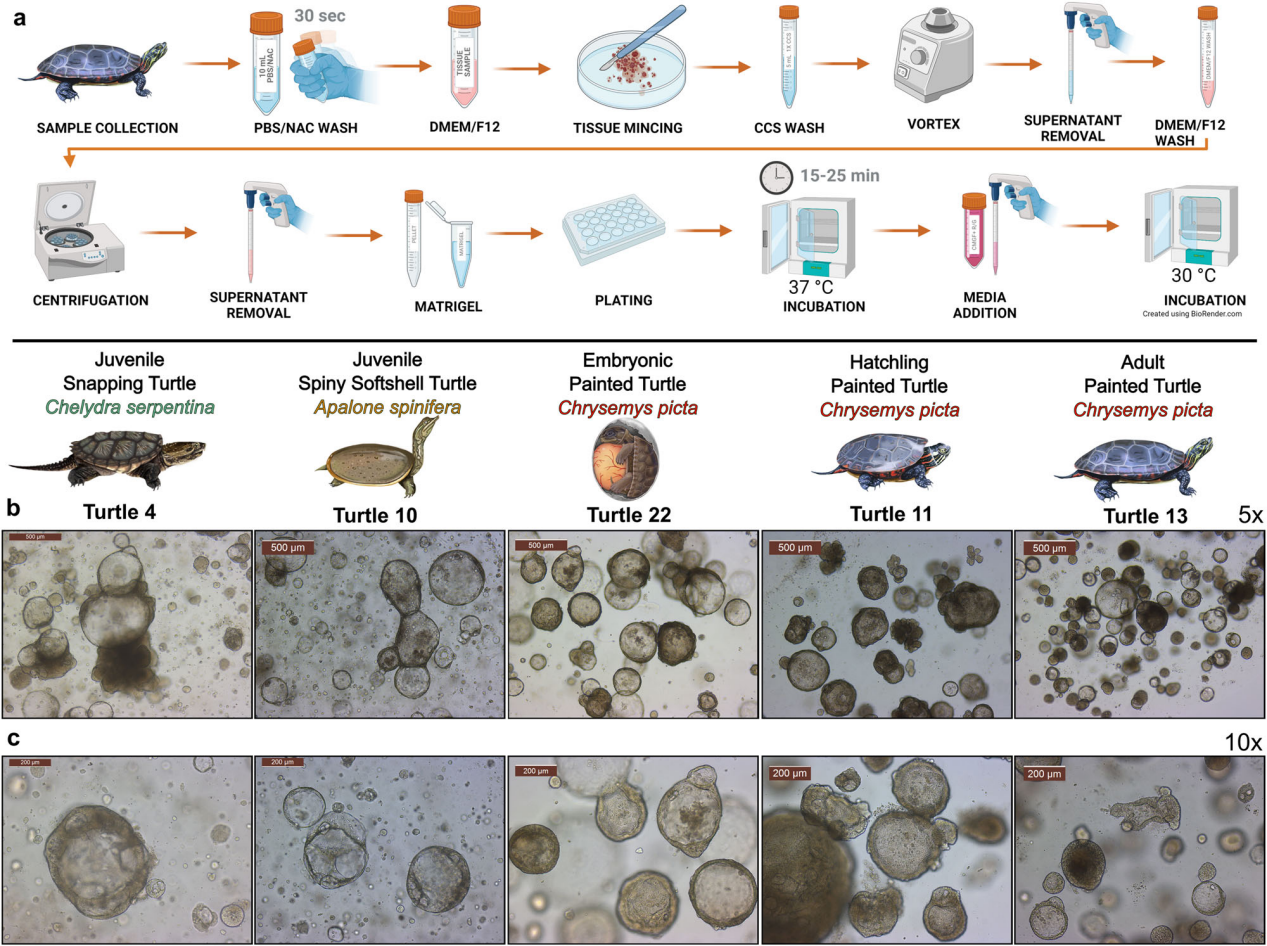
Morphological characterization and isolation optimization for turtle liver organoids. Culture protocol and characterization of turtle liver organoids. **a** Minimal workflow to isolate and culture turtle organoids, from tissue collection, mincing, washing, plating, and incubating the turtle liver tissue. **b**, **c** Light microscopy images of organoids derived from the liver of snapping turtle (*Chelydra serpentina*), spiny softshell turtle (*Apalone spinifera*), as well as embryonic, hatchling, and adult painted turtle (*Chrysemys picta*) at 5X (**b**) and 10X (**c**) magnification. Scale bars are in μm. Created using Biorender.com.

Here we report the culture of turtle-derived organoids from the liver of juvenile spiny softshell turtle (*Apalone spinifera*) and snapping turtle (*Chelydra serpentina*), as well as embryonic, hatchling, and adult painted turtles (*Chrysemys picta*) (Fig. 1). Our optimization of the isolation procedure for turtle liver organoids eliminated unnecessary steps typically followed when attempting to isolate intestinal stem cells^18^, which expedited the final experimental protocol (Fig. 1a) and included media supplementation with prostaglandin E2 (PGE2) at 9.93 µM. A subset of samples were cryopreserved, including liver organoid lines from *C. serpentina* (*n* = 1), and *C. picta* (*n* = 1 embryonic, *n* = 3 hatchling, and *n* = 3 adult) (Supplementary Data 1). Organoid lines withstood multiple passages (Supplementary Fig. 1), with the embryonic organoids of a painted turtle being passaged a total of 14 times across 97 days before cryopreservation without signs of decelerated growth (Supplementary Data 1), suggesting their potential for continuous expansion. The proliferation capability of liver organoids was highlighted by immunohistochemical hybridization of Proliferating Cell Nuclear Antigen (PCNA) (Supplementary Fig. 2). Importantly, six cryopreserved organoid lines were successfully thawed, re-cultured, and expanded, including juvenile *C. serpentina* (*n* = 1), plus embryonic (*n* = 1), hatchling (*n* = 1), and adult (*n* = 3) *C. picta* (Supplementary Data 1). In contrast, although *A*. *spinifera* organoids were successfully isolated during our first few attempts, these organoids typically did not proliferate into larger numbers after the second or third passage as needed. Thus, organoid samples from *A*. *spinifera* were collected solely for histological characterization, while further optimization of media and temperature is warranted to improve their long-term culture in the future.

### Turtle liver morphology

Organoid structures commonly lack terminally-differentiated cells due to the inherent contrasts that exist between in vivo and in vitro environments^39^. Thus, the process of identifying cellular components of organoids in many ways parallels the approaches used to identify undifferentiated cancer cells in pathology where a combination of methods (morphology, transcripts/protein markers, etc.) are often needed^39,40^.

**Fig. 2:**
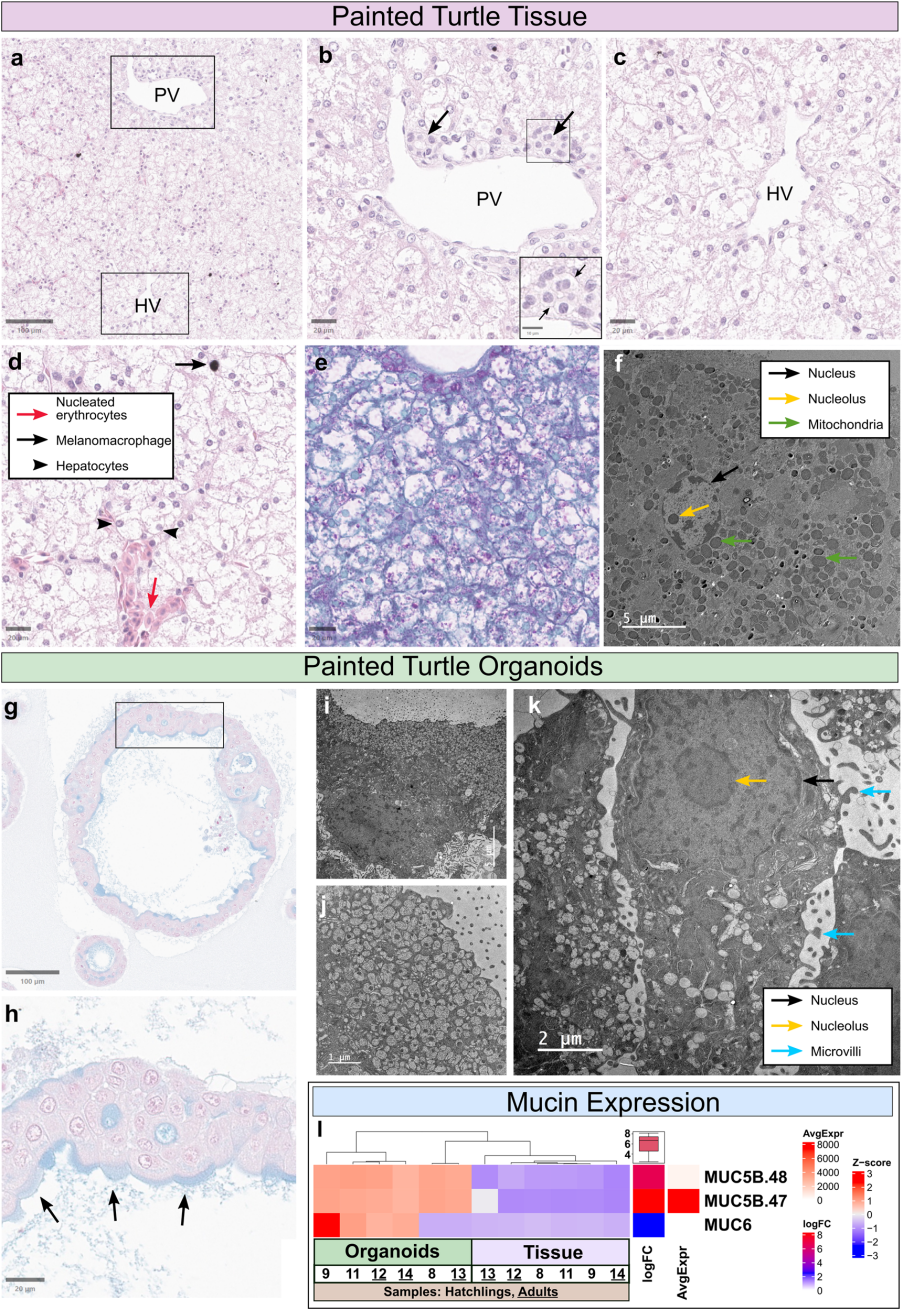
Histopathology staining and transmission electron microscopy of painted turtle liver tissues and organoids. Overview of turtle liver histology from a hatchling painted turtle (*Chrysemys picta*). **a**–**c** Turtle liver lobules were distinguished by a portal venule (Pv) in the peripheral portal triad and the terminal hepatic venule (Hv) centrally in the lobule. In the portal triad, a small bile duct is seen (b, inset and arrows) next to the Pv. **d** Nucleated erythrocytes (red arrows) are seen within sinusoids and melanomacrophages (black arrow) are readily detected in the interstitium by their cytoplasmic pigment. Hepatocytes (black arrowheads) often have round nuclei and the cells are distended by increased rarefaction, which parallels the magenta color of Periodic acid Schiff (PAS)+ glycogen (**e**). **f** Transmission electron microscopy (TEM) of turtle liver tissue with arrows identifying structures of interest (nucleus = black, nucleolus = yellow, green = mitochondria). Note the frequent mitochondria characteristic for hepatocytes. **g**, **h** Alcian blue (AB) histochemical stain of hatchling turtle (*Chrysemys picta*) liver organoids showed AB+ mucins in the apical cytoplasm of epithelioid cells lining the lumina (inset and arrows). Note also the round nuclei that has central to basal localization in the cytoplasm. **i**, **j** TEM images of turtle liver organoids show mucin granules in apical cytoplasm, confirming AB+ staining. **k** TEM of the turtle liver organoids with arrows identifying structures (nucleus = black, nucleolus = yellow, blue = microvilli). Note the relative paucity of mitochondria compared to hepatocytes (see Fig. 2F). Hematoxylin and eosin (HE) stain (**a**–**d**), PAS stain (**e**), Osmium stained TEM (**f**, **i**–**k**) and Alcian blue stain (**g**, **h**). Scale bars are in µm. **l** RNA-seq expression of mucin genes detected in *C*. *picta* organoids and tissues from hatchlings and adults. The main matrix-colored cells are the Z-scores for the normalized count data for gene expression (red = higher expression, blue = lower expression). The boxplot shows the summary of the values for the log Fold Change (logFC) data column. *Hatchling* and *tissue* were set as baseline in the contrasts, therefore blue depicts a bias towards their expression and red depicts a bias towards organoid or adult expression. AvgExpr = Average Expression.

The fundamental organization and structure of the liver in several species including turtles have been described in detail elsewhere^41–43^. Briefly, the turtle liver is organized into lobules that are structurally defined in the periphery by the portal tracts containing the portal venule (Pv), bile duct and hepatic arteriole, and defined centrally by the terminal hepatic venule (Hv) (Fig. 2a–c, Supplementary Fig. 3a–c). At the periphery of the lobule, blood from the portal venule and hepatic arteriole mix in the sinusoidal capillaries and flow towards the hepatic venule along cords of hepatocytes. In a countercurrent flow in the lobule, bile is produced by hepatocytes and secreted into canaliculi that flow peripherally towards the bile ducts of the portal triads^42^. The majority of the liver is composed of epithelial cells, namely hepatocytes and cholangiocytes. Hepatocytes are defined as polygonal and polarized epithelial cells that have abundant mitochondria and centrally oriented nuclei, but whose morphological appearance can vary widely due to their multiple roles in metabolism, detoxification, secretion, and in cellular storage (e.g., glycogen) (Fig. 2d–f, Supplementary Fig. 3d–f). Cholangiocytes are simple cuboidal to columnar epithelial cells lining the biliary duct system and gallbladder that transport and modify bile before it reaches the intestine. Cholangiocytes have protective roles such as mucus secretion to protect the cells against the caustic nature of the bile^44^ and mucus granules can be observed in the apical cytoplasm^45–47^. Some other cells in the turtle liver that can be readily defined histologically, include nucleated erythrocytes within the sinusoidal capillaries, or melanin-containing pigmented phagocytes (known as melanomacrophages)^48^ in the interstitial connective tissue (Fig. 2d, Supplementary Fig. 3d–f). In contrast, other liver cells are difficult to distinguish solely by light microscopy, including fibroblasts, endothelial cells, stellate cells, and resident macrophages (Kupffer cells).

### Morphological organoid characterization

The morphology of all the turtle liver organoids under bright field microscopy mostly resembled cystic spheroids, although darker spheroids with a thicker organoid border were also observed (Fig. 1b, c). Additionally, *A. spinifera* liver organoids typically displayed a thinner organoid border and visible vacuoles in the organoid compared with *C. serpentina* and *C. picta* (Fig. 1c). Histopathologic examination of the turtle liver organoids showed a contiguous simple layer of cuboidal to columnar epithelioid cells lining the cystic spaces, whose morphology resembles that of hepatocytes or cholangiocytes. Further examination revealed that these polarized epithelial cells often had Alcian Blue+ mucus in the apical cytoplasm (seen in *C. picta* hatchling organoids) that was corroborated as electron lucent vacuoles in the apical cytoplasm by transmission electron microscopy (seen in *C. picta* adult organoids). Additionally, these cells had central to basolateral oriented nuclei and contained relative scarce mitochondria (Fig. 2g–k) compared to native hepatocytes (compare to Fig. 2f). Combined, these morphological features are most consistent with cholangiocytes.

### Bulk stranded RNA-seq analysis of hatchling and adult painted turtles from liver organoids and tissue

For *C. picta*, over 94% of RNA-seq reads from each library (tissue and organoids from both hatchlings and adults) mapped as pairs to the reference genome (Chrysemys_picta_BioNano-3.0.4)^7^. For *C. serpentina*, the mapping rate to the painted turtle reference genome was lower (88% of read pairs for the organoid and 84% of read pairs for the tissue), likely due to molecular divergence of their genomes during ~105 million years (My) since their last common ancestor, which impairs cross-species mapping somewhat (Supplementary Data 2). We used the *C. picta* genome as a reference because the existing *C. serpentina* genome assembly^49^ is not fully annotated and is more fragmentary.

**Fig. 3:**
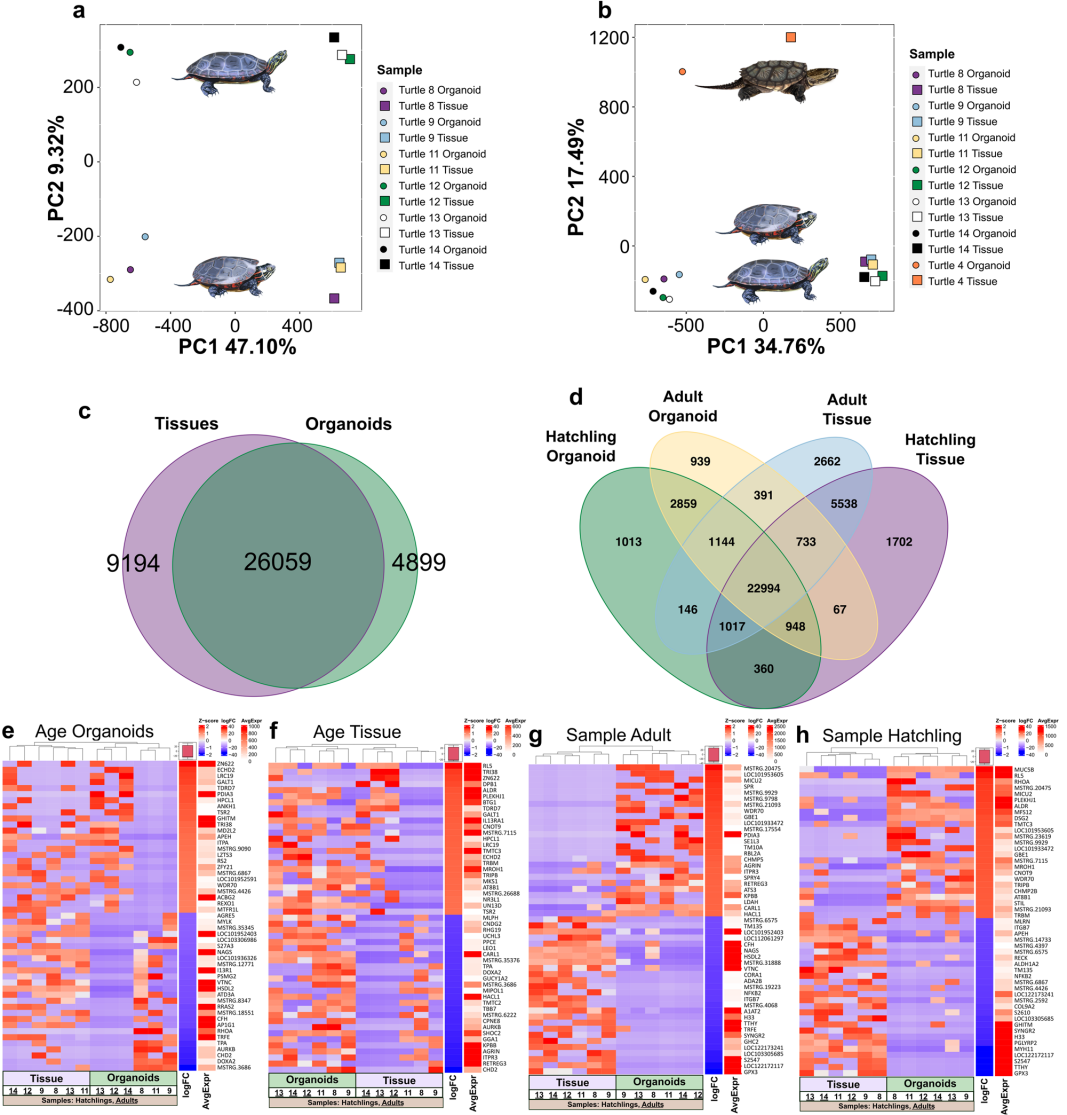
Transcriptomic characterization of turtle liver organoids and the tissue of origin. Transcriptomic expression patterns of liver tissue and organoids. **a** Principal components analysis (PCA) plot displaying clustering of transcriptomes by sample types for *Chrysemys picta* (circles = organoids; squares = tissues) (*C. picta* hatchlings *=* purple, blue, yellow; *C. picta* adults *=* green, white, black). **b** Inclusion of *Chelydra serpentina* (orange) in the PCA plot reveals species-specific differences in genome-wide transcription. **c** Venn diagram illustrates the number of shared and uniquely expressed genes in *C. picta* tissues (purple, *n* = 6) and organoids (green, *n* = 6) combined from hatchlings and adults. **d** Venn diagram illustrating the number of shared and uniquely expressed genes across *C. picta* sample types and life stages (hatchling organoid = green [*n* = 3], adult organoid = yellow [*n* = 3], hatchling tissue = purple [*n* = 3], adult tissue = blue [*n* = 3]). **e**–**h** Heatmaps of differentially-expressed genes (DEGs) in *C. picta* between age groups (hatchling vs. adult) and sample type (organoid vs. tissue). Specifically, heatmaps identified DEGs when comparing (**e**) age within organoids, (**f**) age within tissue, (**g**) sample type in adults, and (**h**) sample type in hatchlings. *Hatchling* and *tissue* were set as baseline in the contrasts therefore blue is a bias towards their expression and red is a bias towards organoid or adult expression. logFC log Fold Change, AvgExpr Average Expression.

A principal component analysis (PCA) of the normalized gene model counts for liver organoid and liver tissue transcriptomes in painted turtles indicated strong clustering by both age and sample type (Fig. 3a), as well as interspecific differences when snapping turtle data were included (Fig. 3b). In *C. picta* alone (Fig. 3a), PC1 primarily captured variation (47.10%) due to sample type (tissue vs. organoid), while PC2 primarily captured variation (9.32%) due to age (hatchling vs. adult). When including *C. serpentina* (Fig. 3b), PC1 still captured variation (34.76%) due mostly to sample type (tissue vs. organoid), a relationship that was retained across species, while PC2 primarily captured variation (17.49%) due to species, and to a lesser degree by age (hatchling vs. adult) when examining *C. picta* clusters.

The transcriptome assembly for painted turtles generated 54,050 initial gene models, which were annotated using several approaches, including: 1) a genome-guided annotation from the assembler and 2) a blastx of all transcripts against the UniProt SwissProt vertebrate database, both of which were followed by 3) a blastn of unannotated transcripts against the RNACentral ncRNA database. Note that the blasts utilized transcript sequences, not gene-level sequences as these are what is produced by the assembly. The best supported transcript annotation (based on bitscore, evalue, and query coverage) of all transcripts assigned to a gene was used to determine the gene-level annotation. From the total, 18532 gene models were successfully annotated both by genome-guided and blastx methods, 6690 only through the genome-guided approach, 10146 only through blastx, and 15199 gene models were redundant and likely represent isoforms, leaving 18682 gene models unannotated by either method, of which 3672 were later identified as ncRNAs. Some of the remainder 15010 unannotated gene models may be noise and not represent true transcripts, but others show high expression and likely represent true but yet-to-be annotated genes. We note two sources of undetectable redundancy in our approach: 1) genome-guided annotations include uncharacterized LOC annotations that may be redundant upon full characterization in the future, and 2) UniProt derived annotations sometimes differ slightly in their gene names from genome-guided gene names depending on the organism of origin (e.g. human IDs vs turtle IDs).

Organoids shared more expressed genes with their tissue of origin than not. A total of 27,876 of the 54,050 initial gene models had a baseMean of >50. After filtering out gene models with less than 100 reads across all 12 libraries, we found >26K genes that were expressed in both liver organoids and tissues, while 4.8K genes were uniquely expressed in liver organoids and 9K in liver tissue when hatchlings and adults were combined (Fig. 3c). Similarly, when data were separated by life stage, the majority of genes (~23K) were expressed in both organoids and tissues of hatchlings and adults, whereas only ~1K genes were uniquely expressed in organoids of any age, and ~1K or ~2.6K genes were uniquely expressed in hatchling or adult tissue, respectively (Fig. 3d). Although most genes were expressed in common, DEGs were detected between organoids and tissues (15,671 in adults and 14,711 in hatchlings). Figure 3e–h illustrates the top 50 most divergently expressed genes between tissues and organoids (either up- [red] or downregulated [blue]). As expected across life stages, differentially expressed genes (DEGs) were detected between adult and hatchling organoids (884 DEGs), and between adult and hatchling tissues (3,839 DEGs).

### Expression profiling of genes of interest in C. picta across life stages

When searching the transcriptomes for genes involved in hepatic function, two important genes were upregulated in *C. picta* hatchling liver organoids compared with adult liver organoids (Supplementary Data 3a, Fig. 3e). The first is tissue-type plasminogen activator (*Tpa*) which is involved in plasminogen conversion into plasmin (a main enzyme responsible for clot breakdown) that is currently used in medical applications, and which was the only previously approved pharmacological treatment for restoring blood flow after a stroke occurred^50^. The second gene is transforming protein RhoA (*Rhoa*), which is involved in a pathway that promotes actin polymerization in the cell cytoskeleton (among other changes) and is thought to play a role in the anoxic overwintering ability of turtles, affecting the actin dynamics in the cell^51^. In contrast, genes that were upregulated in adults compared with hatchling liver organoids included protein disulfide-isomerase A3 (*Pdia3*) and enoyl-CoA hydratase domain-containing protein 2 (*Echd2*), both of which encode proteins secreted from primary human hepatocytes^52^. Additionally, dual oxidase maturation factor 2 (*Doxa2*), was upregulated in hatchling compared with adult liver organoids, which is a gene found to be upregulated in the liver of the Chinese softshell turtle (*Pelodiscus sinensis*) under anoxic conditions, possibly due to increased reactive oxygen species (ROS) or disturbed mitochondrial function^53^.

In liver tissues (Supplementary Data 3b, Fig. 3f), upregulated genes in adult *C. picta* compared with hatchlings included 60S ribosomal protein L5 (*Rl5*), which is associated with the cellular mechanism that is responsible for translating mRNA to proteins^54^. Additionally, protein BTG1 (*Btg1*) was upregulated in adults compared with hatchling tissues, a gene encoding a protein with anti-proliferative function that prevents cell growth and lowers energy demands, which is upregulated in painted turtles under anoxic conditions^55^.

### Differentially expressed genes of interest between C. picta liver organoids and tissues, in hatchlings and in adults

In adult liver organoids, many DEGs were involved in glycogen production or storage (Supplementary Data 3c, Fig. 3g), two important liver functions that regulate energy metabolism. Upregulated genes in adult liver organoids compared with adult liver tissue included 1,4-alpha-glucan branching enzyme 1 (*Gbe1*), a glycogen branching enzyme, and protein disulfide-isomerase A3 (*Pdia3*), which is secreted by hepatocytes^52^ and responds to endoplasmic reticulum stress, helping modulate the folding of newly synthesized glycoproteins^56^. Compared with adult liver tissues, adult liver organoids also showed upregulation of two additional interesting genes. The first is tRNA methyltransferase 10 homolog A (*Tm10a*), a tRNA modification enzyme, that may be involved in the protective stress response^57^. The second is phosphorylase b kinase regulatory subunit beta (*Phkb*; *Kpbb*), which stimulates glycogen breakdown^58^, and predicts poor prognosis in human hepatocellular carcinoma patients when downregulated, whereas it inhibits cell proliferation and induces apoptosis of tumor cells when artificially upregulated^59^.

Finally, several genes were upregulated in hatchling liver organoids compared with hatchling tissues (Supplementary Data 3d, Fig. 3h). These include Mucin-5B (*Muc5b*) (Fig. 2l), a mucin expressed in mammalian cholangiocytes^60^, transforming protein RhoA (*Rhoa*), which is involved in anoxic overwintering in the painted turtle^51^, and 1,4-alpha-glucan branching enzyme 1 (*Gbe1*), a glycogen branching enzyme. Several other DEGs were upregulated in hatchling liver organoids, including protein O-mannosyl-transferase TMTC3 (*Tmtc3*) which, when downregulated, reduces transcripts that are involved in degrading proteins^61^, and CCR4-NOT transcription complex subunit 9 (*Cnot9*), a member of the CCR4-NOT complex which helps maintain liver homeostasis via mRNA deadenylation in order to modulate the liver transcriptome^62^.

### Enrichment analysis

The graphical results from treemap (Supplementary Figs. 4–6) illustrate the similarity and differences in enrichment patterns between all groups in *C. picta* (hatchling tissue, hatchling organoid, adult tissue, adult organoid). The group terms obtained by treemap provide a supercluster representative, describing the general patterns of multiple related clusters of genes. While a deeper examination reveals more granular details of specific terms present, these supercluster terms allow for assisted identification of trends, which were consistent with the role of the liver in maintaining metabolic homeostasis.

*For Biological Process GO terms* (Supplementary Fig. 4), hatchling and adult organoid groups shared the same top three supercluster terms: organonitrogen compound catabolic process, organic substance metabolic process, and nitrogen compound transport, consistent with the role of the liver in protein metabolism^63^. Further, hatchling and adult tissues also shared the organic substance metabolic process with both organoid groups, and adult tissues also shared enrichment of organonitrogen compound catabolic process with hatchling and adult organoids. mRNA metabolic process was a top supercluster term for both hatchling and adult tissues. A third top supercluster term for hatchling tissues was cellular catabolic process.

*For Cellular Component GO terms* (Supplementary Fig. 5), ribonucleoprotein complex was a top supercluster term for all four groups consistent with the importance of ribosome biogenesis to sustain liver function^64^. Adult and hatchling organoids shared the term organelle subcompartment, while hatchling tissues included the term intracellular organelle lumen, and adult tissues included the term organelle membrane and cytosol. The second-tier superclusters for all four groups overlapped in most of their terms including: endomembrane system, intracellular anatomical structure, membrane-enclosed lumen, organelle, and protein-containing complex. Both organoid groups also shared the supercluster term envelope.

*For Molecular Function GO terms* (Supplementary Fig. 6), among top supercluster terms shared by all four groups were mRNA binding, catalytic activity, structural constituent of ribosome, and structural molecule activity. Hatchling and adult organoids also shared the supercluster term hydrolase activity, which is also implicated in liver function and disease [e.g. ref. ^65^]. An additional prominent supercluster term for hatchling organoids was catalytic activity, acting on a nucleic acid.

These biological processes, cellular components, and molecular functions, relate to liver function and exhibit an overall shared pattern of functional enrichment in the top superclusters seen between groups when comparing *C*. *picta* across age (hatchlings and adults) and sample type (organoids and tissues). Differences were also observed in some other clusters, consistent with the notion that organoids are simplified versions of the original tissue. Overall, the similarities in liver-related functions highlight the potential of the turtle liver organoid model as a useful and biologically appropriate in vitro tool for a variety of research topics, keeping in mind that not all cell types are represented in this model compared to the tissue of origin.

### Single-nuclei RNA-seq reveals cell clusters in embryonic liver organoids

**Fig. 4:**
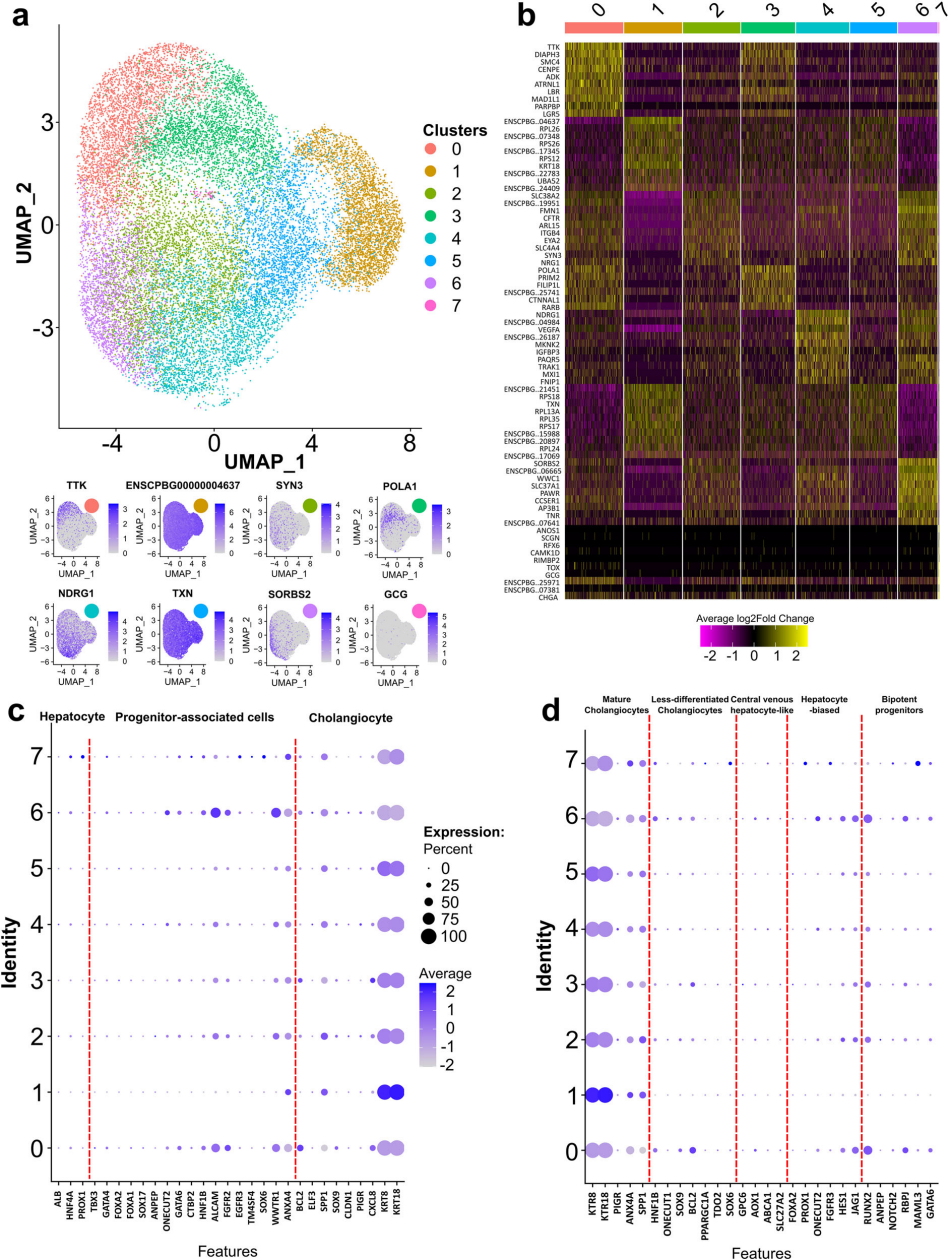
Single-nuclei RNA-seq analysis of embryonic *Chrysemys picta* liver organoids. Identification of cell clusters using snRNA-seq. **a** Unannotated UMAP showing the 8 distinct cell clusters (colored) identified in embryonic *Chrysemys picta* liver organoids, with the transcription profiles (in blue) of eight genes found across clusters (higher intensity = greater expression), expressed as log fold change. **b** Heatmaps identifying up to the top 20 markers for each cluster (upregulated = yellow and downregulated = purple, with respect to each other), expressed as average log2Fold Change. **c** Expression of known genes representing hepatocytes, progenitor-associated cells, and cholangiocytes for each cluster^83^ (separated by red dotted line). **d** Expression of known genes representing mature cholangiocytes, less-differentiated cholangiocytes, central venous hepatocyte-like, hepatocyte-biased, and bipotent progenitor cells for each cluster^83^ (separated by red dotted line). Dot size depicts the percentage of cells in a class and dot color corresponds to the average expression level across all cells within a class (blue = higher transcription, gray = lower transcription).

After transcriptomes of hatchling and adult *C. picta* liver organoids had been characterized, we were successful in growing embryonic turtle liver organoids, whose transcriptome was characterized using single-nuclei RNA-seq (snRNA-seq) to identify relevant cell clusters. This dataset is composed of 387,570,615 sequenced reads derived from 21,384 single nuclei samples with a total of 18,852 features. Overall, 95.3% of the reads mapped to the painted turtle reference genome. A total of 8 distinct cell clusters were identified in the embryonic *C. picta* liver organoids with the most highly expressed gene from each cluster used to visualize expression across all cells (Fig. 4a). Relative expression level across clusters was also compared for the top expressed genes per cluster (Fig. 4b). The transcriptome of each cluster was compared to known liver cell types (Fig. 4c) and specific cholangiocyte cell types (Fig. 4d) to further characterize the expression profile across clusters (Supplementary Data 4). Based on these known cell markers, all clusters had a strong cholangiocyte signature (*Krt8* and *Krt18*) while a subset of cells also had limited expression of markers found in progenitor-associated cells (*Alcam* and *Wwtr1*) (Fig. 4c). Upon further division of specific cholangiocyte cell types, markers for mature cholangiocytes were the most prominent for all clusters (Fig. 4d).

### MS-based proteomic characterization of turtle organoids

**Fig. 5:**
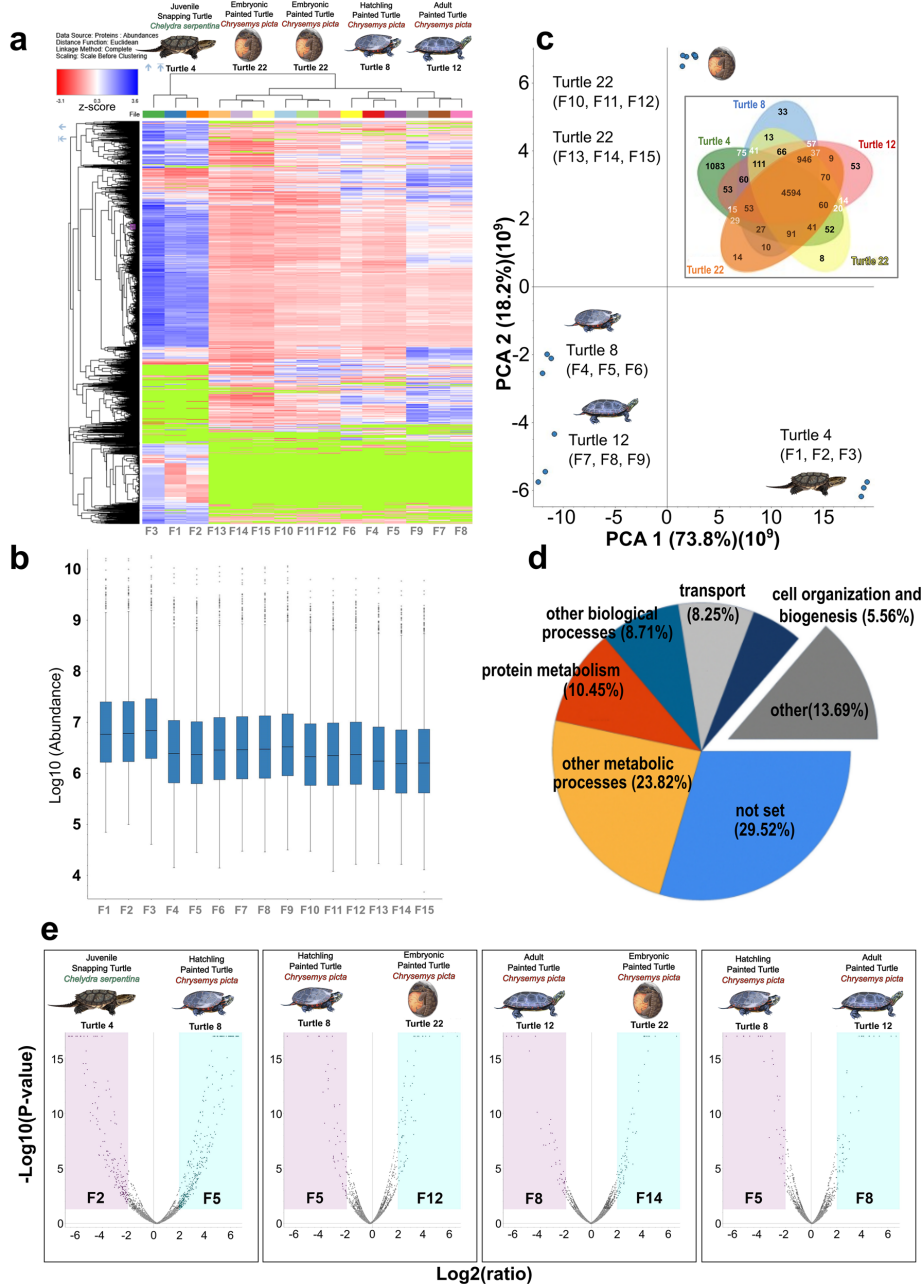
Exploration into the proteins expressed in turtle liver organoids. Proteomic characterization of turtle liver organoids. **a** Heatmap of the differentially expressed proteins (DEPs) (upregulated = blue, downregulated = red) observed between juvenile *Chelydra serpentina* (*n* = 1) and embryonic (*n* = 2), hatchling (*n* = 1), and adult (*n* = 1) *C. picta* liver organoids. Protein expression values were Z-score normalized prior to clustering. Green signifies no matches. **b** Boxplot of the total abundance of proteins for each replicate. Each box represents the distribution of values within a group, with horizontal lines indicating the median (spanning from the 25th to the 75th percentile), error bar lines denoting adjacent values (1.5 interquartile range of the 25th and 75th percentile), and dots marking observations outside the range of adjacent values. **c** PCA plot of the proteomes from the four individuals. Insert contains a Venn diagram of the unique and overlapping proteins detected among juvenile *Chelydra serpentina* and embryonic, hatchling, and adult *C. picta* liver organoids. **d** Pie chart displaying the enriched GO terms for biological processes in organoids. **e** Volcano plots comparing DEPs between a replicate representative per individual from different sample groups (upregulated = blue, downregulated = purple).

To identify proteins enriched in our samples, liquid chromatography followed by mass spectrometry (LC-MS/MS) was carried out on the organoids and comparisons of protein expression across juvenile *C. serpentina* and embryonic, hatchling, and adult *C. picta* liver organoids were made. Because no leftover tissue was available by this time, a proteomic comparison between tissue and organoids was precluded. We applied hierarchical clustering analysis to compare individual proteins identified across all samples and generated a heatmap to illustrate their relative abundance (Fig. 5a) as well as displaying their total abundance (Fig. 5b). A Principal Component Analysis (PCA) of proteomics data (Fig. 5c) depicted the variation in the proteomes among biological and technical replicates, and it indicated clear differences between the proteomic profiles by age and between species. Technical triplicates clustered close to each other, whereas *C. serpentina* triplicates form an outlier cluster on the PCA plot (Fig. 5c), with a similar grouping observed in the Venn diagram (Fig. 5c). Furthermore, after enrichment analysis of the proteome, GO terms related to the proteins found across organoid samples can be seen with the top four terms including “other metabolic processes”, “protein metabolism”, “other biological process”, and “transport” (Fig. 5d). Volcano plots displayed the degree of upregulated and downregulated proteins when comparing samples from two individuals (Fig. 5e). Finally, the proteomic expression of liver specific cell markers was compared to the transcriptomic expression shown in Supplementary Fig. 7. Multiple mature cholangiocyte markers were highly expressed in the transcriptome including *Krt8*, *Krt18*, and *Anxa4*, while in the proteome, KRT8 and KRT18 proteins were also highly expressed in hatchling and adult *C. picta* Supplementary Fig. 7a, b.

## Discussion

Reptiles have lagged behind traditional biomedical research models such as *Caenorhabditis elegans*, *Drosophila melanogaster*, *Danio rerio*, and *Mus musculus*^66^. While there are good reasons for this, including the small size, fast life cycle, and high genetic or physiological similarities to humans of some traditional models^67^, the unique evolutionary adaptations of non-model taxa provide an opportunity to answer fundamental questions that are otherwise difficult to investigate^68^. Indeed, scientific interest in reptilian genomics has expanded greatly since the sequencing of the green anole *Anolis carolinensis*^69^, and the taxonomic scope of studies is growing rapidly from single representatives of major non-avian reptilian lineages thanks to mounting genomic resources^70^.

The development of turtle organoids presented here is the first description for the chelonian Order and second for non-avian reptiles after snakes^28^, to the best of our knowledge, and represents a major step towards building a toolkit that overcomes the technical challenges associated with the slow maturation and seasonal reproduction that characterizes turtles and other long-lived reptiles. Turtle organoids may be used to study a variety of biological processes at the cellular and molecular level, particularly as functional genomic tools become available. Namely, these turtle organoids are amenable to the implementation of techniques such as gene editing using the Clustered Regularly Interspaced Short Palindromic Repeats (CRISPR) system^71,72^, providing a promising alternative for long-lived turtles over the approach used for the first genome editing of a reptile, the brown anole, *Anolis sagrei*^12^. Although successful, these initial editing attempts had a low throughput and required microinjections into immature oocytes of live adult females^12^, which makes large-scale gene editing and screening experiments impractical for turtles. The development of organoids and functional genomic tools in other turtles and reptiles overcome these limitations and will render genetic editing faster, more accessible, more precise, and will be supported by the exponentially growing number of vertebrate genome assemblies^73^ combined with protocols developed here and elsewhere^12,13,28^.

The time course of our optimization of the organoid isolation procedures from fresh turtle liver tissue progressed first using the spiny softshell turtle (*Apalone spinifera*), then snapping turtle (*Chelydra serpentina*), and finally, hatchling, adult, and embryonic painted turtles (*Chrysemys picta*). These turtles are abundant and relatively well-studied, representing emerging models for ecology, evolution, and biomedical research^3,49,74^. Turtle liver organoids, which were characterized and compared to the tissue of origin at the morphological and transcriptomic levels, with additional organoid proteomics, bolster the promise of these emerging species by providing an in vitro system that adds to mouse^75^, human^76^, and canine hepatic organoids, which were successfully cultured to model human diseases^23^.

Some important modifications to our previously described canine organoid isolation protocol^18^ are worth noting. The five 1X Complete Chelating Solution (CCS) washes and ethylenediaminetetraacetic acid (EDTA) incubation to separate cells from intestinal tissue and to discard the microvilli^18^, were not required for turtle organoids, as inclusion of the tissue aided in initial turtle organoid growth. Therefore, our approach represents the minimal effective protocol to successfully grow turtle organoids, as described in the methods section. Overall, our optimized protocol allows for expedited isolations of more samples at a lower cost while minimizing potential contamination. Incubation temperature for organoids must also follow species-specific requirements, which for the turtles studied here was 30 °C, as has previously been described for 2D turtle fibroblast cultures of the same taxa^9^, whereas mammalian organoids are typically cultured at 37 °C, and snake venom gland organoids at 32 °C^28^. As previously mentioned, *A. spinifera* liver organoids were difficult to expand in culture which led to a lower passage number for these organoid lines than for the other species. Hence further refinement of the protocol, media, or incubation temperature is needed to improve the yield of liver organoids for this species.

Because many turtle organoids exhibited spaces/vacuoles in their structure which might decrease the total cell number per organoid, to increase the successful passaging and recovery rate for turtles, turtle organoids were allowed to grow larger than in protocols previously reported for canine hepatic organoids prior to passaging. The expansion capability of our organoids was underscored by their successful passages over 14 times prior to final cryopreservation without signs of decelerated growth during that time, and was also supported by the positive immunostaining using the Proliferating Cell Nuclear Antigen (PCNA) marker. Similar staining was reported for mouse liver ductal organoids^77^, mouse epithelial organoids^78^, and paraffin-embedded human liver organoids, indicating their highly proliferative nature^79^. Of note, some turtle liver tissues displayed dark pigmentation of the cells, which resemble melanomacrophages^80^. Initially, these melanocytes were thought to be caused by contaminants (such as bacteria and fungi) in the turtle liver organoid cultures in our study. However, the pigmented cells disappeared when the leftover initial tissue was removed from the culture. Lastly, adding Prostaglandin E2 (PGE2) to the media for the culture of *C. serpentina* (*n*=2), as done for snake venom gland organoids^28^, allowed for successful cultivation of *C. serpentina* liver organoids, and PGE2 was therefore included in all subsequent cultures of turtle liver organoids. Further investigation into the effect and necessity of PGE2 is warranted.

Transcriptomic analysis permitted a more in-depth molecular characterization of the turtle organoids than by interrogating only a few liver markers, as is commonly done. This approach showed that turtle organoids expressed the majority of genes in common with their tissue of origin, underscoring that they were derived from liver tissue. Yet, as expected, tissues also tended to have more unique genes expressed, likely reflecting the greater complexity of cell types present in them compared with organoids. Indeed, differences in gene expression between organoids and the tissue of origin have been observed in other studies using transcriptomics, e.g. in snake venom gland organoids^28^, and canine intestinal organoids^20^, among others. The turtle liver organoids generated here are epithelial in origin and currently lack cell types such as immune cells, but emerging methods exist to co-culture immune cells with organoids^81^ that may be used to create a more complex model. In our hatchling samples, *Muc5b* was the most extreme DEG between liver organoids and tissues, likely because our media composition seems to encourage mucin production. Cholangiocytes produce apical mucins in pig tissues and organoids^47^, and *Muc5b* and *Muc6* are the main mucin genes^82^. Consistently, *Muc5b* and *Muc6* expression was detected in the transcriptome of turtle liver organoids, further supporting the histology and TEM characterization which identified a subset of cells in the *C. picta* hatchling organoids that secreted mucin.

Further characterization of our embryonic *C. picta* liver organoid model included snRNA-seq, which allowed a single-cell level resolution precluded with bulk RNA-seq. When compared to published single-nucleus and single-cell RNA-seq data of human liver tissue^83,84^ our snRNA-seq revealed expression of mature cholangiocyte markers and lower expression in some clusters of some progenitor-associated cell markers. Moreover, our findings agree with snRNA-seq expression in human hepatic organoids that also exhibit a strong cholangiocyte signature, and revealed upregulation of a mucin gene (*Muc13*) in 3D compared to 2D cultures^84^. In our bulk RNA-seq analysis, overlap of multiple liver specific genes was seen between tissues and organoids. The snRNA-seq data show that small cell populations strongly expressed markers of other cell types in the organoids, a detail which is masked in bulk RNA-seq. Future studies should analyze turtle liver tissue using snRNA-seq to determine its cellular heterogeneity. Additionally, future experiments could include specific growth factors to enhance the differentiation of the organoid culture towards a hepatocyte lineage^75^, which will expand the applicability of these turtle liver organoids. This is particularly promising because embryonic cholangiocytes in mammals can function as liver progenitor cells (LPCs) that may differentiate into hepatocytes, cholangiocytes, and LPCs [reviewed in ref. ^85^]. Both transcriptomic and proteomic data revealed distinct differences in DEGs and DEPs between snapping (*C. serpentina*) and painted (*C. picta*) turtles, uncovering species-specific molecular architecture in their liver organoids. Proteomic analysis also revealed shared expression of proteins across the three life stages of *C. picta*. Such divergence between ages illustrated in our PCA plots was similarly observed in previous transcriptomic studies of various tissues in *C. picta* and other turtles, across embryos, hatchlings, and adults^74,86–90^. Proteomic results coincided with our transcriptomic data in revealing the prevalent expression of cholangiocyte-specific cell markers in the liver organoids, consistent with the cholangiocyte-enriched organoids from human liver^84^ as well as others^91,92^. Again, mammalian adult cholangiocytes can transdifferentiate into hepatocytes in vitro and likely in vivo [reviewed in ref. ^85^], and our turtle organoids open the door to test whether the same is true in reptiles.

While this study focused on the establishment and characterization of turtle liver organoids, mechanistic tests to fully evaluate their potential as relevant models in biomedicine and other biological areas are still needed, as described below. Future studies should also include characterization steps throughout the growth of the liver organoids, or after the thawing of samples, which was not conducted here. The next research phase should include further long-term growth, testing the potential differentiation of cell types and their subsequent characterization, which would add to the utility of these turtle liver organoids.

Turtle species can be useful biomedical models to investigate their unique adaptive strategies to overwintering, such as supercooling and anoxia tolerance^33^, as well as their extended life spans^93^. 3D organoids provide a novel genomic resource to study these remarkable adaptations, and the characterization of these organoid lines is needed prior to experimentation, including testing for their ability to withstand supercooling and survival in hypoxic conditions as live turtles do. Here, we utilized MS-based proteomics for characterization and stranded mRNA expression analysis which revealed key differences between hatchling and adult *C. picta* liver organoids, including genes related to iron-binding proteins, antioxidant proteins, and serpins, which are upregulated in the liver of hatchling painted turtle in response to freezing and anoxic conditions^33^. Turtle organoids could potentially accelerate the development of turtles as a relevant biomedical model to improve human survival after stroke or heart attack, liver organ preservation techniques for transplantation surgery, and tissue preservation during hypoxia and anoxia.

Our transcriptomic and proteomic comparison should serve as a baseline for future experiments when attempting to differentiate hepatocytes in these cultures to study the ability of organoids to metabolize compounds. Additionally, our preliminary characterization of protein expression serves as a baseline resource to identify candidate proteins that might regulate the unique adaptations of turtle liver cells underlying the overwintering potential and supercooling ability of turtles, which has major biomedical implications to improve human liver organ preservation before transplantation. Additionally, as *C. picta* is an ideal model for studying the ability of cells to survive hypoxic conditions, these liver organoids may serve as a useful model for future identification of proteins deployed in tissue preservation during hypoxia and anoxia.

Our first success culturing turtle liver organoids opens the door to apply this technology to derive turtle organoids from other tissues and species as done by our group and others [e.g. refs. ^28,94^], which is essential for evolutionary developmental biology and other comparative studies. For example, this emerging technology holds promise to illuminate the molecular basis of temperature-dependent sex determination (TSD), which is urgent in the face of climate change, as TSD turtle embryos develop into males or females based on ambient temperature. Indeed, the rapid increase of average temperatures on Earth threatens critical biological processes and global biodiversity^95^. Among many detrimental effects, warmer temperatures can alter sex ratios. These skewed ratios have already threatened the viability of natural populations of several sea turtles (all TSD)^96,97^, and a recent model predicts imminent feminization of some sea turtle populations^98^. Yet, the molecular basis of TSD has not been fully elucidated, despite the recent discovery of genetic and epigenetic candidates (reviewed in ref. ^99^). Our protocol to culture turtle organoids from *C. serpentina* (TSD) and three life stages of *C. picta* (TSD), as well as *A. spinifera*, a turtle with a ZZ/ZW sex chromosome system of genotypic sex determination (GSD)^100^, should be applicable, with some modification, to culture turtle organoids from other tissues, such as gonads, to decipher the mechanisms and pathways underlying turtle sex determination.

Organoids have immense potential in other areas of biology. For instance, over half of all known turtle species (>50%) of data-sufficient taxa are threatened^1^, which limits and precludes deciphering their unique biology, evolution, and potential for biomedical research. Namely, studying endangered species can be challenging due to obstacles such as permit requirements, small population sizes, and low reproducibility of results, among others^101^. Being able to create a reusable in vitro model is therefore critical because species protection laws that protect endangered species also hinder the study of the molecular basis of their adaptations that could be essential to conservation efforts. These limitations can be overcome by leveraging organoid technology, a scientific toolkit that adheres to the 3Rs principles of humane animal experimentation^102^ minimizing the need for tissue sampling and helping overall species survival.

Future studies can use this in vitro turtle organoid model to assess the viability of each turtle life stage when exposed to different stressors, such as environmental toxins or xenobiotics, by using LIVE/DEAD viability and cytotoxicity staining^103,104^. Additionally, ecotoxicological studies can be strengthened by studying organoids of sentinel turtle species that are sensitive to environmental pollutants found in their habitats. For example, sea turtle cells were used to identify potential novel biomarkers of chemical exposure, including annexin^4^. Turtle-derived organoids could potentially be a relevant biological model to identify such biomarkers in vulnerable populations, as they better represent the tissue of origin compared with conventional primary 2D cell lines. One such important environmental pollutant in freshwater ecosystems is cadmium which can be ingested by turtles. Cadmium affects hepatic enzyme levels, gene expression, and DNA methylation in turtles, thereby exhibiting toxic damage to the liver^8,105,106^. Therefore, we hypothesize that turtle-derived liver organoids could potentially be a valuable model for studying aquatic ecosystem health and further cellular and molecular effects of heavy metal pollution on endangered and non-vulnerable species alike.

The creation and preliminary characterization of turtle liver organoid lines derived from three different species of turtles, opens the door for genetic manipulation within a major vertebrate clade that is understudied due to their slow maturation, seasonality, lack of functional genomic resources, and prevalent endangered status. This work lays a path for turtle organoids to now be tested in functional assays and determine their relevance in applications including ecologic toxicology, supercooling/cryopreservation, and anoxia/hypoxia research, as well as many other ecological and evolutionary studies. Our study expands the application of organoid technology across the Tree of Life, facilitating future study of adaptations in reptiles and other non-model species relevant to the broad biological and biomedical communities.

## Methods

### Animal husbandry and tissue collection

Five juvenile spiny softshell turtles (*Apalone spinifera*), three juvenile snapping turtles (*Chelydra serpentina*), plus two embryonic, three hatchling, and three adult painted turtles (*Chrysemys picta*) were used in this study. Animals were collected in Iowa under appropriate permits from the Iowa DNR (SC648 and SC595), and all procedures followed protocols approved by the Institutional Animal Care and Use Committee (IACUC) (IACUC-21-121) of Iowa State University as described below. Details of the donor animals, including sex, age, and the outcome of the organoid culture, can be found in Supplementary Data 1. Adult males and freshly laid *C. picta* eggs were collected from the wild. Hatchlings and juveniles were obtained from eggs incubated in the laboratory at 26 °C (*C. picta*), a temperature that produces exclusively males in painted turtles, as this species displays temperature-dependent sex determination (TSD) and lacks sex chromosomes^107^. The embryonic sample at developmental stage 22^108^, was obtained from a *C. picta* egg incubated at 26 °C. Eggs of *C. serpentina* (TSD) were incubated at 27.5 °C, which produces a mixed sex ratio^109^, such that sex of snapping turtle juveniles was diagnosed by gonadal inspection. In contrast, *A. spinifera* displays a ZZ/ZW sex chromosome system of genotypic sex determination (GSD)^100^ and produces both sexes at 27.5 °C^110^, the temperature used here to incubate their eggs. Thus, *A. spinifera* juveniles can be sexed by PCR amplification of sex-linked markers^111^, a simpler method than by qPCR of rDNA repeats^112^.

Live animals were housed indoors in water tubs, provided with UV A/B bulbs and a dry surface for basking, and kept at ~24 °C until processing. Animals were fed Tetra ReptoMin sticks *ad libitum*. Animals were euthanized, then washed in iodine and hydrogen peroxide, and sex was diagnosed (*C. serpentina*) or confirmed (*C. picta*) by gross gonadal morphology or presumed by the incubation temperature (*C. picta* embryo). Tissues were quickly harvested inside a biosafety cabinet, and a subset of tissue was immediately placed into RNAlater (Invitrogen; AM7021) (except embryonic liver) and another in formalin, for downstream processing.

### Organoid culture

Experimental methods for liver organoid isolation followed our previously published canine protocol^18^ with minor modifications. The formulation of expansion media (*Complete media with growth factors with ROCK inhibitor and GSK3β inhibitor - CMGF+ R/G*) and the optimization and variations of the organoid isolation protocol are listed in Supplementary Data 5 and 1 respectively. The optimized and minimum required protocol for liver organoid isolation consisted of rinsing the fresh tissue in Phosphate Buffered Saline (PBS)/N-acetylcysteine (NAC) once and then transferring it to a tube filled with Advanced DMEM/F12 (Gibco; 12634-010), mincing the tissue into small fragments, washing in complete chelating solution (1X CCS) once, vortexing of the sample, removing the supernatant, adding 6 mL of DMEM, centrifuging at 100 × *g* (700 × *g* was used for the first samples, but was lowered to 100 *×* *g* to assist in the separation of dead cells or debris) for 5 min at 4 °C, removing the supernatant, mixing the pellet of cells and tissue fragments (to aid in initial growth) with Matrigel® Matrix (Corning; 356231, 356255), subsequent plating in 24 well plates (Corning; 3524), and adding media (Fig. 1a). Because embryonic *C. picta* liver tissue was smaller than at later life stages, the protocol was further shortened to submersion in DMEM, centrifuging at 100 *×* *g* for 5 min at 4 °C, removal of supernatant, addition of 200 µL DMEM, centrifuging again, mincing, transferring to a tube and centrifuging, then mixing with Matrigel and plating. Prostaglandin E2 (PGE2) (Tocris; 2296) 9.93 µM was added to the culture media, as was previously done for snake venom gland organoids^28^. When passaging or cleaning, organoids were resuspended in Matrigel and solidified at 37 °C and 5% CO_2_ in an incubator (PHCBi; MCO-170ML-PA) for ~15–30 min to avoid organoid damage at warmer temperatures. The passaging technique used consisted of adding 500 µL of TrypLE™ Express (Gibco; 12604-021) to ~500 µL of organoids resuspended in DMEM at 37 °C for 10 min, with gentle flicking halfway through. When culturing embryonic liver organoids and re-growing frozen samples, if organoids would not pellet, the addition of 2 mL of Cell Recovery Solution (Corning; 354270) to 1 mL of organoids suspended in DMEM and a subsequent incubation on ice for 10 min assisted in degrading excess Matrigel. After spinning, 6 mL of DMEM was used to wash away the Cell Recovery Solution before plating. Additionally, if samples previously resisted passaging when given TrypLE™ Express, samples were passed in Cell Recovery Solution as opposed to DMEM which typically dissociated the organoids into single cells or small clusters. Organoids were incubated in a Nuaire Direct Thermal incubator at 30 °C and 5% CO_2_. A few cultures had a subset of wells incubated at 37 °C to try and assist in expansion, however this was unsuccessful and no wells incubated at 37 °C were used for downstream analysis (Supplementary Data 1).

### Cryopreservation

Organoids were frozen in alternative freezing media, including (1) 50% CMGF + R/G + PGE2, 40% FBS, and 10% DMSO as well as (2) Cryostor CS10 (BioLife Solutions; 210102) to test their thawing potential. Before freezing, organoids were resuspended in freezing media, placed overnight at −80 °C in a Mr. Frosty container (Nalgene; 5100-0001) filled with isopropanol, and then transferred to liquid nitrogen (−196 °C) indefinitely.

### Transmission electron microscopy

Expanded organoids had media removed, and were transferred to a 15 mL tube using Cell Recovery Solution, placed in ice for ~10 min to degrade Matrigel, centrifuged at 100 or 700 × *g* at 4 °C for 5 min, the supernatant was discarded, the pellet was resuspended in 1% paraformaldehyde and 3% glutaraldehyde in phosphate-buffered saline (PBS) and stored at 4 °C prior to processing. The samples were fixed for 48 h at 4 °C in 1% paraformaldehyde, 3% glutaraldehyde in 0.1 M sodium cacodylate buffer, pH 7.2. After washing in a cacodylate buffer 3 times for 10 min each, samples were post-fixed with 1% osmium tetroxide in 0.1M sodium cacodylate buffer at room temperature for 1 h. Next, the samples were washed with deionized water 3 times for 15 min each, and then *en bloc* stained using 2% uranyl acetate in distilled water for 1 h. Then the samples were washed for 10 min in distilled water, dehydrated for 1 h in each step of a graded ethanol series (25, 50, 70, 85, 95, 100%), dehydrated further with 3 changes of pure acetone for 15 min each, and then infiltrated with EmBed 812 formula (hard) for EPON epoxy resin (Electron Microscopy Sciences, Hatfield, PA) with graded ratios of resin to acetone until fully infiltrated with pure epoxy resin (3:1, 1:1, 1:3, pure) for 6–12 h per step. The tissues were then placed into BEEM capsules and polymerized at 70 °C for 48 h. Then, using a Leica UC6 ultramicrotome (Leica Microsystems, Buffalo Grove, IL), 1.5 µm thick sections were made and stained with EMS Epoxy stain (a blend of toluidine blue-O and basic fuchsin). Thin sections were made at 50 nm and collected onto single slot carbon film grids. Finally, TEM images were captured using a 200 kV JEOL JSM 2100 scanning transmission electron microscope (Japan Electron Optics Laboratories, Peabody, MA) with a GATAN One View 4K camera (Gatan inc., Pleasanton, CA).

### Histological stains

Tissue samples were fixed in 10% formalin while organoids had their media removed and 500 µL of Formalin-acetic acid-alcohol (FAA, composition in ref. ^18^) was added to wells containing Matrigel. Both tissues and organoids were changed to 70% ethanol 24 h later prior to paraffin-embedding. Tissue and organoids were stained with hematoxylin and eosin (H&E), Alcian Blue, and Periodic acid–Schiff (PAS) at the Iowa State University Histopathology Department. After staining, slides were scanned on Leica Aperio GT 450 Scanner and analyzed with ImageScope (v12.4.3.5008) to characterize the morphology and cell type composition of tissues and organoids. For immunohistochemistry, slides were stained for proliferating cell nuclear antigen (PCNA) (DAKO; 0879) at a 1:400 dilution, and images were taken on an ECHO Revolution microscope (ECHO).

### RNA extractions

Expanded organoids were collected using one of two methods: (1) being transferred to a 15 mL tube with Cell Recovery Solution, placed in ice for ~10 min to degrade Matrigel, centrifuged at either 100 or 700 *×* *g* at 4 °C for 5 min, supernatant was removed, then resuspended in Advanced DMEM/F12, centrifuged, and supernatant discarded, or (2) being transferred to a 15 mL tube with Advanced DMEM/F12, centrifuged at either 100 or 700 *×* *g* at 4 °C for 5 min, supernatant was removed, then resuspended in Cell Recovery Solution, placed in ice for ~10 min to degrade Matrigel, centrifuged, and supernatant discarded. After procedure (1) or (2) above, the pellet was then resuspended in 100 µL of PBS and transferred to a cryovial. A volume of 900 µL of RNAlater was used to flush the 15 mL tube and then added to the cryovial before storage in either liquid nitrogen (−196 °C) or a −80 °C freezer. Tissue biopsies were stored in liquid nitrogen or at −80 °C in cryovials containing 1 mL of RNAlater. After thawing, tissues were rinsed in PBS to remove excess RNAlater solution, immediately transferred into 800 µL of Trizol (Invitrogen; 15596026) and homogenized with a pestle. Organoids were thawed, transferred to a 15 mL tube containing 2 mL of PBS (Corning; 21-040-CM) and centrifuged at 1200 *×* *g* at 4 °C for 5 min to pellet. Excess RNAlater was removed, 1 mL of Trizol was added to the organoids, and then briefly vortexed (5–10 s).

Homogenized samples were stored at room temperature for 5 min, then centrifuged for 10 min at 12,000 *×* *g* at 4 °C to eliminate debris and polysaccharides, whereas the supernatant was transferred to a new tube. Chloroform (Alfa Aesar; J67241) was added (0.2 mL chloroform per mL Trizol), and samples were vigorously shaken for 20 s and incubated at room temperature for 2-3 min. Samples were centrifuged at 10,000 *×* *g* for 18 min at 4 °C, and the aqueous phase was transferred into a new sterile 1.5 mL RNase-free tube. Then, an equal volume of 100% RNA-free EtOH was slowly added using a pipette, mixed, then the samples were transferred to a Qiagen RNeasy column (RNeasy Mini kit; Qiagen; 74104) seated in a collection tube, and centrifuged for 30 s at 8000 *×* *g*, after which the flow-through was discarded and the Qiagen DNase treatment protocol was followed. Next, 500 µL of buffer RPE was added and samples were centrifuged for 30 seconds at 8000 *×* *g*. After discarding the flow-through, another 500 µL of buffer RPE was added and samples were centrifuged for 2 min at 8000 *×* *g*. Flow-through was discarded, and columns were centrifuged for 1 min at 8000 × *g* to remove the remaining buffer. RNA was eluted in 50 µL of RNase-free water (Sigma; W4502-50ML) and allowed to sit for 2 min before centrifuging for 1 min at 8000 *×* *g*. Samples were centrifuged again at 8000 *×* *g*, immediately analyzed on a Nanodrop ND-1000 Spectrophotometer (Thermo Fisher Scientific), and stored at −80 °C.

### RNA sequencing

RNA samples were shipped to GENEWIZ for analysis as follows. The Qubit 2.0 Fluorometer (ThermoFisher Scientific, Waltham, MA, USA) was used to quantify RNA concentration, and a 4200 TapeStation (Agilent Technologies, Palo Alto, CA, USA) was used to measure RNA integrity. Next, an External RNA Control Consortium (ERCC) RNA Spike-In Mix kit (ThermoFisher Scientific; 4456740) was added to normalize the total RNA prior to library preparation. A strand-specific RNA sequencing library was prepared using NEBNext Ultra II Directional RNA Library Prep Kit for Illumina (NEB, Ipswich, MA, USA). Then, the enriched RNAs were fragmented at 94 °C for 8 min. First-strand and second-strand cDNA were subsequently synthesized, with the second strand of cDNA marked by incorporating dUTP during the synthesis (which quenched the amplification of the second strand, helping preserve the strand specificity). cDNA fragments were adenylated at 3' ends, and an indexed adapter was ligated to cDNA fragments, with a limited cycle PCR being used for library enrichment. The sequencing library was then validated on the Agilent TapeStation and quantified using a Qubit 2.0 Fluorometer in addition to quantitative PCR (KAPA Biosystems, Wilmington, MA, USA). The sequencing libraries were multiplexed and clustered onto two flow cells, loaded onto an Illumina HiSeq 4000 instrument, according to the manufacturer’s instructions, and sequenced using a 2x150bp Paired-End (PE) configuration. The HiSeq Control Software (HCS) was used to conduct image analysis and base calling. Raw sequence data (.bcl files) generated from Illumina HiSeq were converted into fastq files and de-multiplexed using Illumina bcl2fastq 2.20 software with one mismatch allowed for index sequence identification.

### Transcriptome assembly

Reads were trimmed with trimmomatic (version 0.39)^113^ to remove low-quality bases and adapter contamination. Reads were checked post-trimming with FASTQC (v 0.11.7)^114^ to confirm quality. Following trimming, reads were mapped to the *C. picta* RefSeq genome (Chrysemys_picta_BioNano-3.0.4)^7^ using GSNAP (version 2021-03-08)^115,116^. Read representation was calculated using samtools (version 1.10)^117^. Following mapping, individual library BAM files were genome-guided assembled with StringTie (version 1.3.4a)^118^ and then merged into a single assembly using the --merge function. Following merging, transcript abundances were calculated for each library and counts were extracted using the prepDE.py script.

### Spike-in and differential expression analysis

In parallel, ERCC reads were mapped to the ERCC reference following the same assembly pipeline as for the sample reads, except that discovery of novel transcripts was not permitted during assembly. Counts for the ERCC transcripts were appended to the gene count matrix for *C. picta*. Differential expression of gene models was calculated with DESeq2 (version 1.24.0)^119^ for *C*. *picta* in R (version 4.0.2)^120^, testing for the effect of age, sample type (adult organoid [*n* = 3], hatchling organoid [*n* = 3], adult tissue [*n* = 3], hatchling tissue [*n* = 3]), and their interaction via a full factorial generalized linear model (Y ~ Age * Sample Type). Estimation of size factors was based on ERCC spike-in transcripts for normalization of the data. As many genes showed an interaction effect, the full factorial model was retained. Differentially expressed genes were filtered based on a baseMean (mean of the counts for all samples that have been normalized for sequencing depth) of >50 and a *P*-adjusted value <0.05. Multiple comparisons were corrected with the FDR/Benjamini–Hochberg method.

### Annotation and enrichment analysis

Blastx (blast-plus version 2.7.1)^121^ against the Uniprot database (accessed May 24, 2022)^122^ was used to further annotate transcripts that were not annotated during the initial genome-guided assembly, although some transcripts remained unannotated after this blastx. Transcript sequences were extracted from the transcriptome using gffread (v0.12.7)^123^. These transcripts were then translated into peptide sequences using TransDecoder (version 5.5.0; https://github.com/TransDecoder/TransDecoder). These sequences were then searched against the PANTHERDB (v17.0)^124,125^ hidden Markov models to obtain compatible sequence identifiers for enrichment analysis. Stringtie transcript counts were converted to gene-level lengthScaledTPM using the tximport package (v1.18.1)^126^ in R. Genes were then mapped to their corresponding PANTHER IDs via transcript isoforms. In the case where multiple isoforms were present, the isoform with the best supported PANTHER ID was prioritized. Unannotated transcripts were filtered out of the analysis, as required by the program.

PANTHER IDs and corresponding expression values were submitted to pantherdb.org for statistical enrichment analysis (Released 2022-10-17) which uses a Mann–Whitney U test to calculate enrichment of GO terms. Enrichment analysis was performed for each library and was searched against the following databases (v17.0 Released 2022-02-22)^127,128^: Pathways, GO-Slim Molecular Function, GO-Slim Biological Process, GO-Slim Cellular Component, and Protein Class. GO terms were filtered for terms that were over-enriched (as opposed to under-enriched). Following GO filtering, terms were filtered for those present in all three biological replicates. These over-enriched and replicated terms were input into REVIGO^129^ for visualization of GO terms after redundancy was reduced (by considering semantic similarity and identification of terms that are most representative of clusters of related terms), using a cutoff of 0.5 and providing terms with FDR values. Obsolete terms were removed, and the dataset was compared to the Whole Uniprot Database and the SimRel semantic similarity measure was used. Analyses were run on 2022/12/09 and the databases used for reference were go.obo (2022-11-03) and goa_uniprot_gcrp.gaf.gz (2022-09-16). Treemap^130^ was used to visualize the resulting clusters. The REVIGO-provided Rscript was downloaded and used to generate plots for interpretation.

### Harvesting for single-nuclei RNA-seq

For single-nuclei processing, organoids were harvested from a sample that had undergone multiple passages but was not previously frozen. Media was removed and 500 µL of Cell Recovery Solution was added to each well, mixed, then placed in ice for 20 min to degrade the Matrigel. The sample was centrifuged at 100 *×* *g* for 5 min at 4 °C, the supernatant was removed, and the pellet was washed with approximately 6 mL of DMEM and centrifuged at 100 *×* *g* at 4 °C for 5 min. The organoid pellet was resuspended in 500 µL of DMEM and transferred to a cryovial. The cryovial was centrifuged at 100 *×* *g* at 4 °C for 5 min, all supernatant was removed, and the pellet was flash frozen in liquid nitrogen for approximately 30 seconds. The sample was then immediately stored at −80 °C, shipped on dry ice the following day to Azenta (South Plainfield, NJ, USA), and stored at −80 °C prior to processing as described below.

### Nuclei isolation

Nuclei extraction was performed using the Miltenyi Nuclei Extraction Buffer (Miltenyi Biotec, Auburn, CA, USA) following manufacturer’s guidelines with gentle MACS Dissociation and C tubes. Upon isolation, the nuclei were counted using trypan blue with a Thermo Fisher Countess III automated cell counter.

### 3’ RNA library preparation and sequencing

Single nuclei RNA libraries were generated using the Chromium Single Cell 3’ kit (10X Genomics, CA, USA). Loading onto the Chromium Controller was performed to target capture of ~10,000 GEMs (‘Gel bead-in-EMulsion’) per sample for downstream analysis and processed through the Chromium Controller. Quality of the sequencing libraries were evaluated on the Agilent TapeStation, then quantified using a Qubit 2.0 Fluorometer (Invitrogen, Carlsbad, CA). Prior to loading onto an Illumina sequencing platform, pooled libraries were quantified using qPCR (Applied Biosystems, Carlsbad, CA, USA). The samples were sequenced at a configuration compatible with the recommended guidelines outlined by 10X Genomics. Raw sequence data (.bcl files) were converted into fastq files and de-multiplexed using the 10X Genomics’ cellranger mkfastq command. Subsequent UMI and cell barcode de-convolution along with mapping to the reference genome Chrysemys_picta_bellii-3.0.3 (GCA_000241765.2) were performed using 10X Genomics Cell Ranger 6.0.1^131^ software package to generate the final digital gene expression matrices and cloupe files.

### Analysis of single-nuclei RNA sequencing data

snRNA-seq reads were mapped to the reference genome (Chrysemys_picta_BioNano-3.0.4) as done for the bulk RNAseq data, and then the Seurat package (v. 4.0)^132^ in R (v. 4.2)^120^ was used. The percentage of reads mapping to mitochondrial genes was used to minimize mitochondrial contamination typically seen in low quality or dying cells, and those with a high percentage were filtered out. Cells were filtered to retain those with gene counts between 200 and 6000 and having less than 40% mitochondrial contamination. Next, the data was normalized using the log Normalization method. Then features with high cell to cell variation were identified. The data was then scaled so that the mean expressions across the cells were 0 and variance across the cells was 1 prior to performing a principal component analysis. Clustering of samples utilized the K-Nearest Neighbor (KNN) method, and using default parameters, clusters were identified.

### Organoid preparation for protein extraction

Media was removed from the culture wells for each sample, then each well of organoids was resuspended in 500 µL of Cell Recovery Solution and pooled into a 15 mL tube before being placed in ice for up to 10 min to degrade Matrigel, and centrifuged at 100 *×* *g* for 5 min at 4 °C. The supernatant was removed, and the pellet was washed with 6 mL of PBS and centrifuged at 100 *×* *g* at 4 °C for 5 min. After one more wash with PBS, the organoid pellet was resuspended in 700 µL PBS and transferred to a cryovial. The cryovial was then spun at 100 *×* *g* at 4 °C for 5 min, all supernatant was removed, and the pellet was flash frozen in liquid nitrogen for ~30 seconds. One cryovial for Turtle 4 (16 wells), Turtle 8 (16 wells), and Turtle 12 (15 wells), and two cryovials for Turtle 22 (10 wells each) were obtained as described, immediately stored at −80 °C, shipped on dry ice, and processed at the University of Texas Rio Grande Valley School of Medicine (UTRGV-SOM).

### Protein sample preparation and trypsin digestion

Cells were lysed to extract proteins using the filter aided sample preparation (FASP) procedure^133^ of the FASP protein extraction kit (ab270519, Abcam)^134^ following the manufacturer’s protocol. Briefly, protein extract was mixed with 200 µL of urea sample solution in a spin filter, centrifuged at 14,000 × *g* for 15 min, and the flow-through was discarded. Next, 10 µL of iodoacetamide solution (10x) and urea sample solution at a 1:9 ratio was added to the mixture, which was vortexed for 1 min and then incubated for 20 min in the dark without mixing. The sample was then centrifuged at 14,000 *×* *g* for 10 min. Then, 100 µL of urea sample solution were added to the spin filter followed by centrifugation at 14,000 *×* *g* for 10 min. Digestion solution (enzyme-to-protein ratio 1:100) was added and vortexed for 1 min and incubated overnight at 37 °C. Next, 50 µM of ammonium bicarbonate solution was added to the spin filter followed by centrifugation at 14,000 *×* *g* for 10 min. After this, 500 µM sodium chloride solution was added to the spin filter followed by centrifugation at 14,000 *×* *g* for 10 min. The filtrate was then acidified with 1 mL of 0.1% trifluoracetic acid (TFA). The extract was then fractionated by using the C18 cartridge solid phase extraction (SPE) for MS analysis. Briefly, the cartridge was conditioned by passing conditioning solution (90/10 MeOH/water with 0.1% TFA v/v/v) through the packing bed. Then, the cartridge was equilibrated by passing equilibration/load solution (0.1% TFA in water v/v) though the packing bed. This was followed by slowly loading the sample into the cartridge packing and passing an additional 1 mL of equilibration/load solution into the cartridge packing. The sample was then desalted by passing desalting solution (5% MeOH/water with 0.1% TFA v/v/v) through the packing bed. The sample was eluted by passing 1 mL of elution solution (50/50 Acetonitrile/water with 0.1% TFA v/v/v) through the packing bed in a new centrifuge tube after allowing complete flow-through of the sample. Lastly, the samples were dried completely in a vacuum centrifuge and stored at −80 °C.

### Proteomic analysis by liquid chromatography and double MS (LC–MS–MS)

After drying, 1 µg of each dried peptide sample was dissolved in 20 µL of 0.05% trifluoroacetic acid with 3% (vol/vol) acetonitrile. In total, 8 µL of each sample was injected into an Ultimate 3000 nano UHPLC system (Thermo Fisher Scientific, Vantaa, Finland). Next, peptides were directly injected and separated on a 15 cm column packed with ReproSil Saphir 1.8 µm C18 beads (Dr. Maisch GmbH, Ammerbuch, Germany). The mobile phase buffer was comprised of 0.1% formic acid in ultrapure water (buffer A) with an eluting buffer of 0.1% formic acid in 80% (vol/vol) acetonitrile (buffer B) ran with a linear 120 min gradient of 6–30% buffer B at flow rate of 300 nL/min. Samples were measured through LC–MS/MS in triplicate (i.e. three 8 µL aliquots from each individual turtle were analyzed) such that Turtle 4, Turtle 8, and Turtle 12 each had 3 technical replicates, whereas Turtle 22 had two sets of triplicates (because 2 samples were processed from Turtle 22 as an extra test of intraindividual replicability of the proteomic analysis). Proteomic data from each replicate was obtained individually and kept separate in downstream analyses. The ultra-high-performance liquid chromatography (UHPLC) was coupled online with an Orbitrap Exploris 480 mass spectrometer (Thermo Fisher Scientific) which was operated in the data-dependent mode, in which a full-scan MS (from m/z 375–1500 with the resolution of 60,000) was followed by MS/MS of the 20 most intense ions (30,000 resolution; normalized collision energy—28%; automatic gain control target (AGC)—2E4: maximum injection time—200 ms; 60 s exclusion). Then, the raw files were directly searched against several turtle protein datasets, including the *Platysternon megacephalum* (big-headed turtle-protein count 21,000), *Terrapene carolina triunguis* (Three-toed box turtle-protein count 31,610), *Pelusios castaneus* (West African mud turtle-protein count 26,820), *Chrysemys picta bellii* (Western painted turtle-protein count 37,046), *Chelydra serpentina* (Snapping turtle-protein count 28,613), *Pelodiscus sinensis* (Chinese softshell turtle-protein count 20,509), and *Chelonia mydas* (Green sea-turtle- protein count 18,960), available in UniProt with no redundant entries, using the Byonic (Protein Metrics) and SEQUEST search engines loaded into Proteome Discoverer 3.0 software (Thermo Fisher Scientific)^135^. MS1 precursor mass tolerance was set at 10 ppm and MS2 tolerance was set at 20 ppm. The search criteria included a static carbamidomethylation of cysteines (+57.0214 Da) and variable modifications of oxidation (+15.9949 Da) on methionine residues and acetylation (+42.011 Da) at N-terminus of proteins. The search was performed with full trypsin/P digestion and allowed a maximum of two missed cleavages on the peptides analyzed from the sequence database with the false-discovery rates of proteins and peptides being set at 0.01. All protein and peptide identifications were grouped, additionally any redundant entries were removed. Only unique master proteins and unique peptides are reported.

### Proteomic data acquisition, quantification, and bioinformatics

After proteomic analysis, all data were quantified using the label-free quantitation node of Precursor Ions Quantifier through the Proteome Discoverer 3.0 (Thermo Fisher Scientific, Vantaa, Finland). For all the quantification of proteomic data calculations, the intensities of peptides were extracted with initial precursor mass tolerance set at 10 ppm, fragment mass tolerance at 0.02 Da, minimum peak count as 1, maximum RT shift as 5 min, PSM confidence FDR of 0.01 as strict and 0.05 as relaxed, with hypothesis tests of difference in the abundance of proteins within and between animals using *t*-test (background based), protein abundance based ratio calculation, 100 as the maximum allowed fold-change, and site probability threshold of 75. To calculate protein abundance, the abundance of all peptide isoforms and fragments identified as belonging to a given protein (i.e. a protein group) in each replicate we added, and this sum was used for downstream analysis, with maximum RT shift of 5 min, pairwise ratio-based ratio calculation, and the maximum allowed fold change set at 100. Additionally, the abundance levels of all proteins and peptides within each replicate were normalized using the total peptide amount normalization node in the Proteome Discoverer 3.0^135^. To calculate fold changes between protein groups, total protein abundance values from all peptide isoforms and fragments of a given protein were added together, and then the ratios of these sums were used to compare proteins among replicates within different samples. Biological significance was inferred for ratios showing a 2-fold change (ratio ≥2.0 or ratio ≤0.5). All visualizations of volcano plots (up and down regulated proteins), heatmap (relative abundance and clustering), pie charts (biological process) and box plots (protein abundances) were created with Proteome Discoverer 3.0^135^. Venn diagrams were created with BioTools.fr^136^.

### Statistics and reproducibility

The study characterized multiple organoid lines and corresponding tissues from multiple species and ages. All figures include labels that identify samples used for each comparison. For bulk RNA-seq, statistical analysis and graphical representation of the data were performed using R (version 4.0.2) using the DESeq2 package (version 1.24.0). Differential expression tests were carried out on samples with three biological replicates for each category and utilized a full factorial model, testing for differences in sample type (organoid vs. tissue) and sample age (adult vs. hatchling). Size factors for expression tests were estimated relative to an ERCC spike-in. Differentially expressed genes were filtered on a *P*-adjusted value < 0.05 utilizing the FDR/Benjamini–Hochberg method to correct for multiple comparisons. snRNA-seq utilized R (v. 4.2) with specific parameters for both snRNA-seq and proteomic comparisons mentioned in the respective methods sections. The figure legends give full information about the number of independent biological replicates (n) analyzed.

Enrichment analysis was performed using length scaled transcript per million (TPM) values as input. A Mann–Whitney U test was used to test for significant enrichment and the False Discovery Rate correction was applied to correct for multiple comparisons. Enrichment analysis was performed independently on each library. Terms that were significant in all three biological replicates were retained for downstream analysis.

### Reporting summary

Further information on research design is available in the Nature Portfolio Reporting Summary linked to this article.

### Supplementary information

Supplementary Information

Description of Additional Supplementary Files

Supplementary Data 1

Supplementary Data 2

Supplementary Data 3

Supplementary Data 4

Supplementary Data 5

Reporting Summary

## Data Availability

The stranded mRNA raw RNA-seq reads and snRNA-seq reads generated and analyzed in this study are available in the Sequence Read Archive (NCBI-SRA BioProject PRJNA931617). The proteomics data are available on the PRIDE database for both *Chelydra serpentina* (Accession PXD048526) and *Chrysemys picta* (Accession PXD048455).

## Code availability

Bioinformatic scripts are available on GitHub (https://github.com/ValenzuelaLab/ZdyrskiEtAl2023_CommBiol_TurtleLiverOrganoids) and: 10.5281/zenodo.10359908^137^.
