# Supplementary material for: Establishment and characterization of turtle liver organoids provides a potential model to decode their unique adaptations
Source: Commun Biol. 2024 Feb 22;7:218. doi: 10.1038/s42003-024-05818-1 (PMC10883927; doi:10.1038/s42003-024-05818-1)
Supplement: Supplementary file 1 — Supplementary Information [file 42003_2024_5818_MOESM1_ESM.pdf]

Communications Biology
SUPPLEMENTARY INFORMATION

Establishment and Characterization of Turtle Liver Organoids Provides a Potential Model to Decode their Unique Adaptations

Christopher Zdyrski * [1,2,11], Vojtech Gabriel [1], Thea B. Gessler [3], Abigail Ralston [2], Itzel Sifuentes-Romero [3], Debosmita Kundu [4], Sydney Honold [1], Hannah Wickham [1], Nicholas E. Topping [3], Dipak Kumar Sahoo [5], Basanta Bista [3], Jeffrey Tamplin [6], Oscar Ospina [7], Pablo Piñeyro [8], Marco Arriaga [9], Jacob A. Galan [9], David K. Meyerholz [10], Karin Allenspach [1,2,5,11], Jonathan P. Mochel [1,2,11], Nicole Valenzuela * [3]

[1] SMART Pharmacology, Department of Biomedical Sciences, Iowa State University, Ames, IA, USA
[2] 3D Health Solutions Inc., Ames, IA, USA
[3] Department of Ecology, Evolution, and Organismal Biology, Iowa State University, Ames, IA, USA
[4] Department of Statistics, Iowa State University, Ames, IA, USA
[5] Department of Veterinary Clinical Sciences, Iowa State University, Ames, IA, USA
[6] Department of Biology, University of Northern Iowa, Cedar Falls, IA, USA
[7] Department of Biostatistics and Bioinformatics, Moffitt Cancer Center, Tampa, FL, USA
[8] Veterinary Diagnostic Laboratory, Iowa State University, Ames, IA, USA
[9] Department of Human Genetics, University of Texas Rio Grande Valley, Brownsville, TX, USA
[10] Department of Pathology, University of Iowa, Iowa City, IA, USA
[11] SMART Pharmacology, Precision One Health Initiative, University of Georgia, Athens, GA, USA

*** Corresponding authors:**

Christopher Zdyrski (czdyrski@uga.edu)

Nicole Valenzuela (nvalenzu@iastate.edu)

This file contains:

- Supplementary Fig. 1
- Supplementary Fig. 2
- Supplementary Fig. 3
- Supplementary Fig. 4
- Supplementary Fig. 5
- Supplementary Fig. 6
- Supplementary Fig. 7

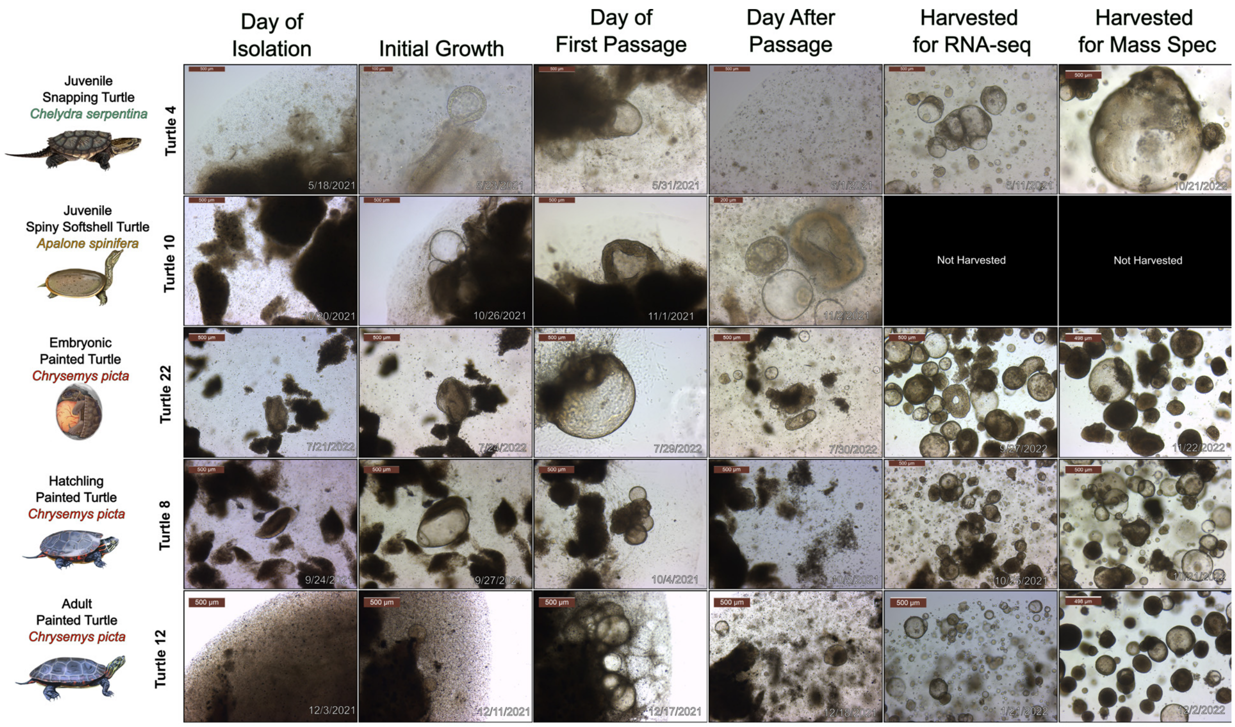

Morphogenesis of turtle liver organoids.

Supplementary Fig. 1: Light microscopy images of turtle liver organoids derived from *Chelydra serpentina*, *Apalone spinifera*, as well as embryonic, hatchling, and adult *Chrysemys picta*. Images spanned from the day of isolation, through a passage, to RNA-seq and mass spectrometry harvesting. Organoids in the “Harvested for Mass Spec” column were from thawed organoid lines, and exhibited no difference in growth compared to lines prior to freezing. Scale bars are in μm.

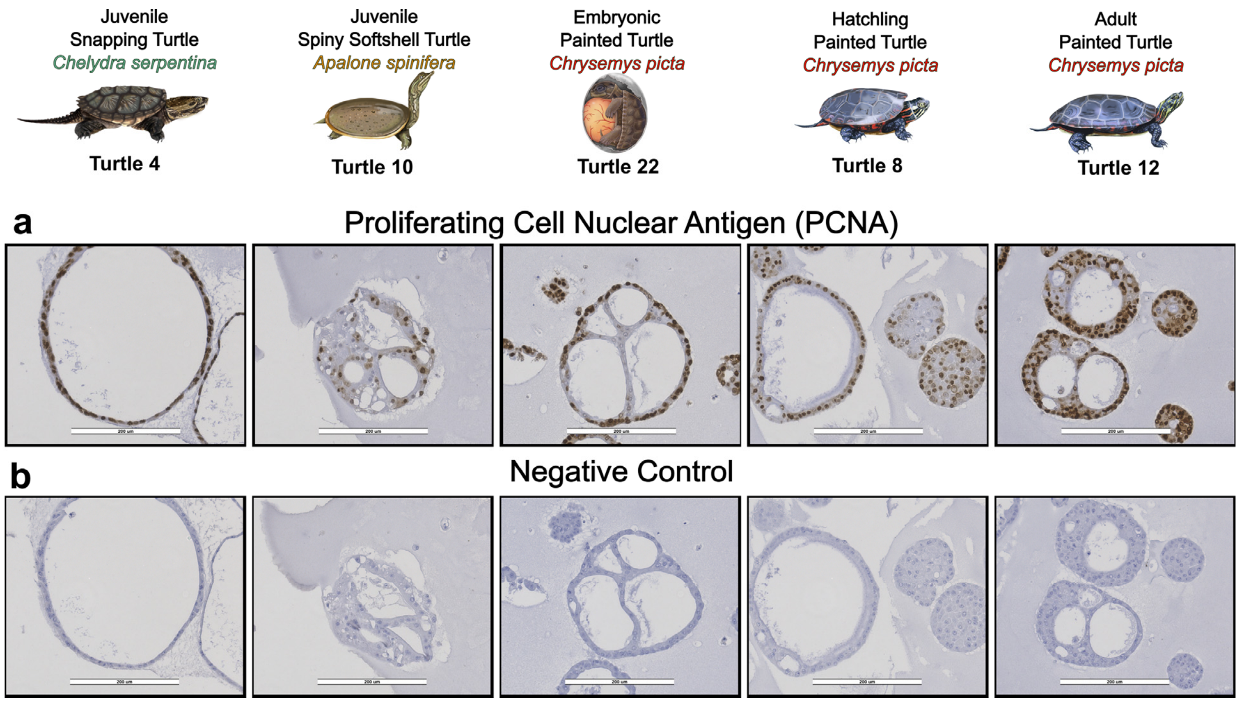

Proliferation of turtle liver organoids.

Supplementary Fig. 2: **(a)** Immunohistochemistry of proliferating cell nuclear antigen (PCNA) and **(b)** negative control images of turtle liver organoids derived from *Chelydra serpentina*, *Apalone spinifera*, as well as embryonic, hatchling, and adult *Chrysemys picta*. Scale bars are in µm.

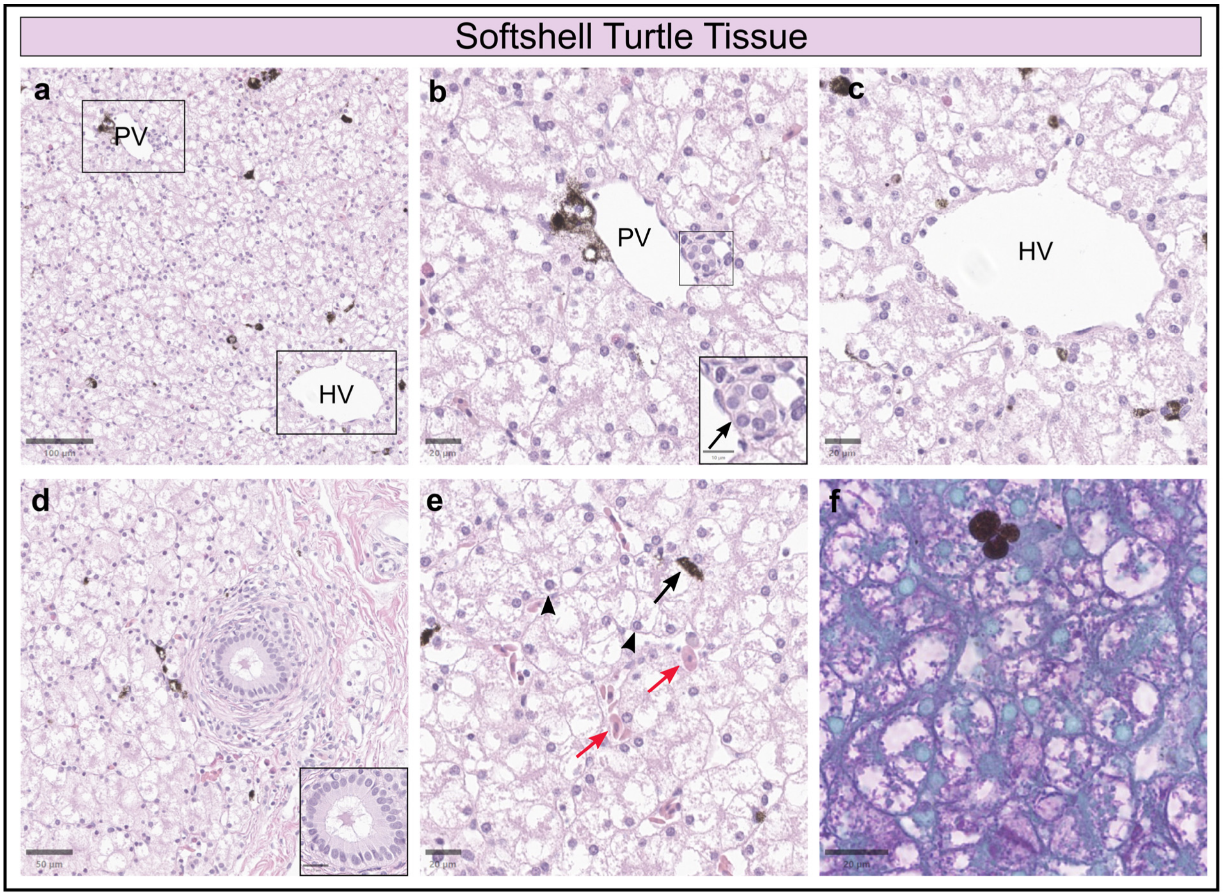

Histopathology staining of softshell turtle liver tissues.

Supplementary Fig. 3: Overview of turtle liver histology from the spiny softshell turtle (*Apalone spinifera*). **(a-c)** Turtle liver lobules were distinguished by the portal venule (Pv) in the peripheral portal triad and the terminal hepatic venule (Hv) centrally in the lobule. In the portal triad, a small bile duct is seen (b, inset and arrow) adjacent to the Pv. **(d)** Note the large intrahepatic bile duct lined by simple columnar and polarized cholangiocytes with basolateral nuclei (inset). **(e)** Nucleated erythrocytes (red arrows) are seen within sinusoids and melanomacrophages (black arrow) are readily detected by their cytoplasmic pigment. Hepatocytes often have round nuclei that are central to basolaterally located in the cytoplasm. Hepatocytes (black arrowheads) are distended and have increased rarefaction of the cytoplasm (e), which parallels the magenta coloration of PAS+ glycogen **(f)**. Hematoxylin and eosin (HE) stain (a-e), PAS stain (f). Scale bars are in μm.

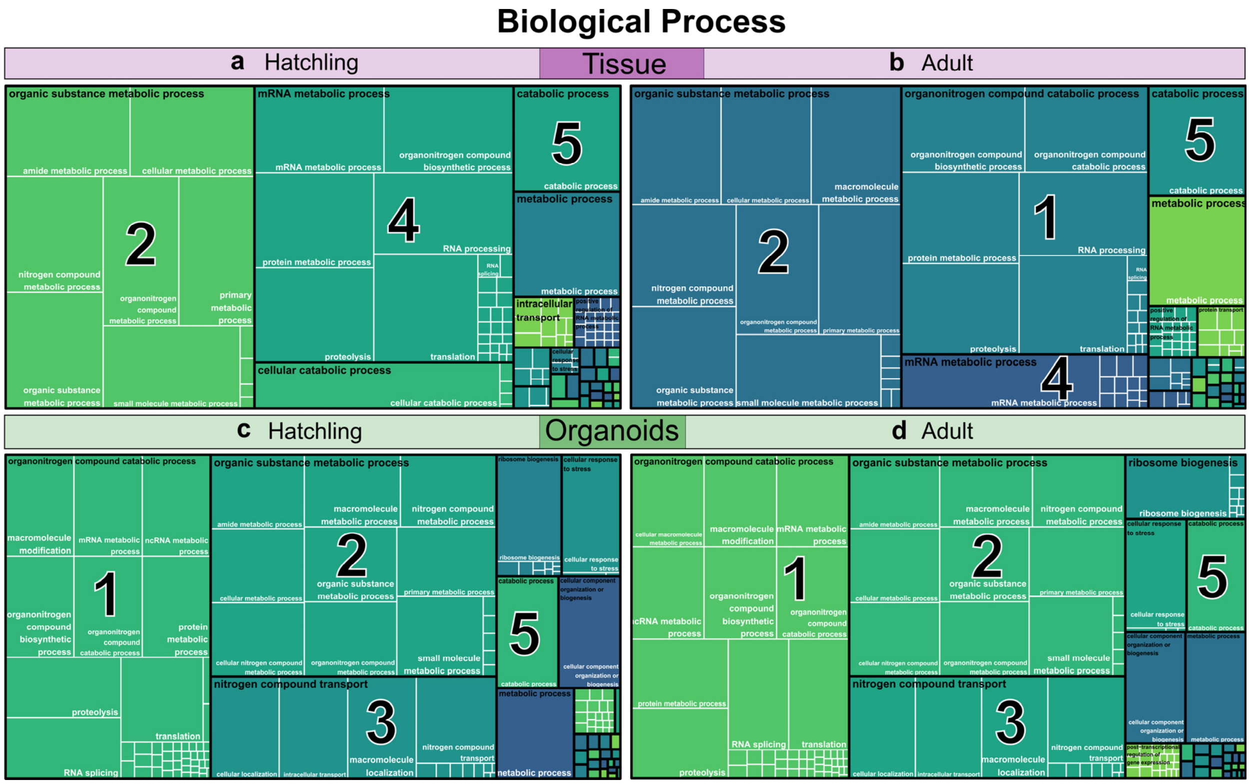

Biological Processes of turtle liver tissue and organoids.

Supplementary Fig. 4: The Biological Processes identified when analyzing the transcriptomic expression patterns of liver tissue and organoids. Enrichment analysis of **(a)** hatchling tissue (*n*=3), **(b**) adult tissue (*n*=3) samples of liver in addition to liver cultures of **(c)** hatchling organoid (*n*=3), **(d)** adult organoid (*n*=3) of *C. picta* liver samples. Top supercluster terms: (1) organonitrogen compound catabolic process, (2) organic substance metabolic process, (3) nitrogen compound transport, (4) mRNA metabolic process, (5) catabolic process.

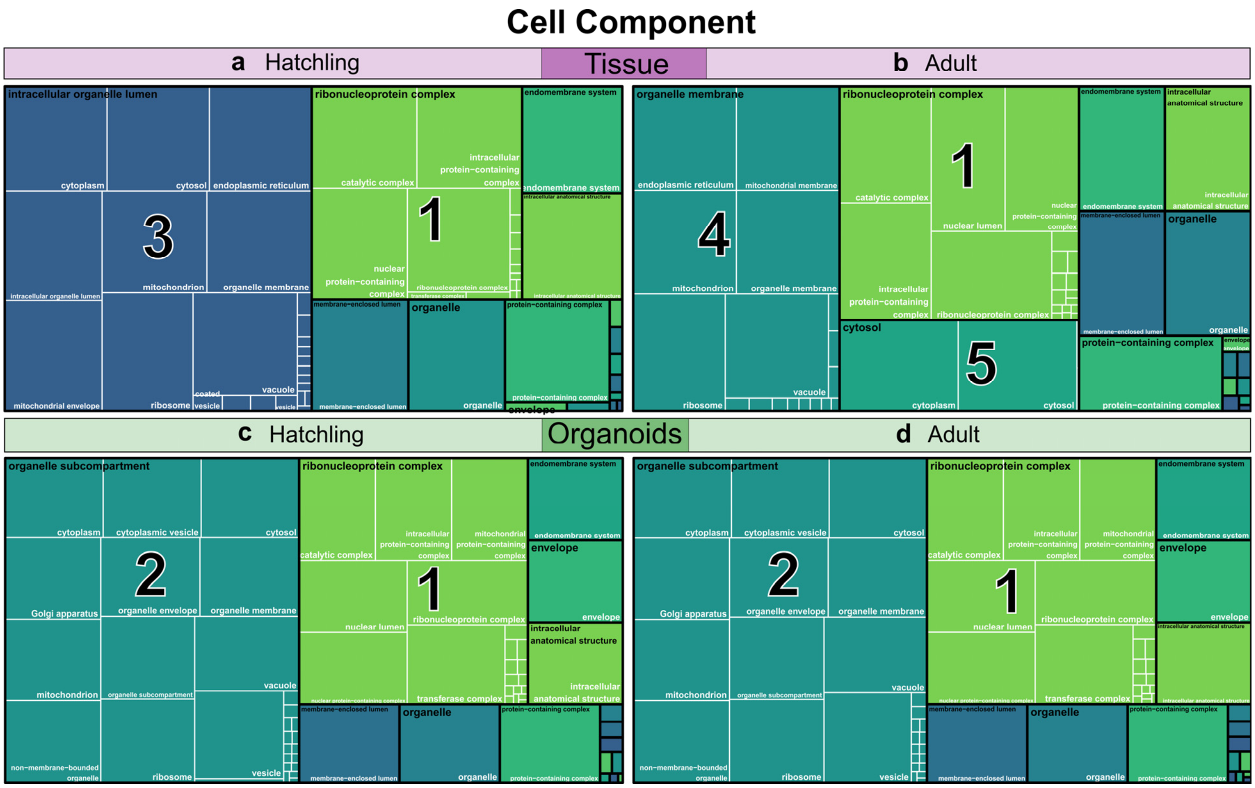

Cellular Components of turtle liver tissue and organoids.

Supplementary Fig. 5: The Cellular Components identified when analyzing the transcriptomic expression patterns of liver tissue and organoids. Enrichment analysis of **(a)** hatchling tissue (*n*=3), **(b)** adult tissue (*n*=3) samples of liver in addition to liver cultures of **(c)** hatchling organoid (*n*=3), **(d)** adult organoid (*n*=3) of *C. picta* liver samples. Top supercluster terms: (1) ribonucleoprotein complex, (2) organelle subcompartment, (3) intracellular organelle lumen, (4) organelle membrane, (5) cytosol.

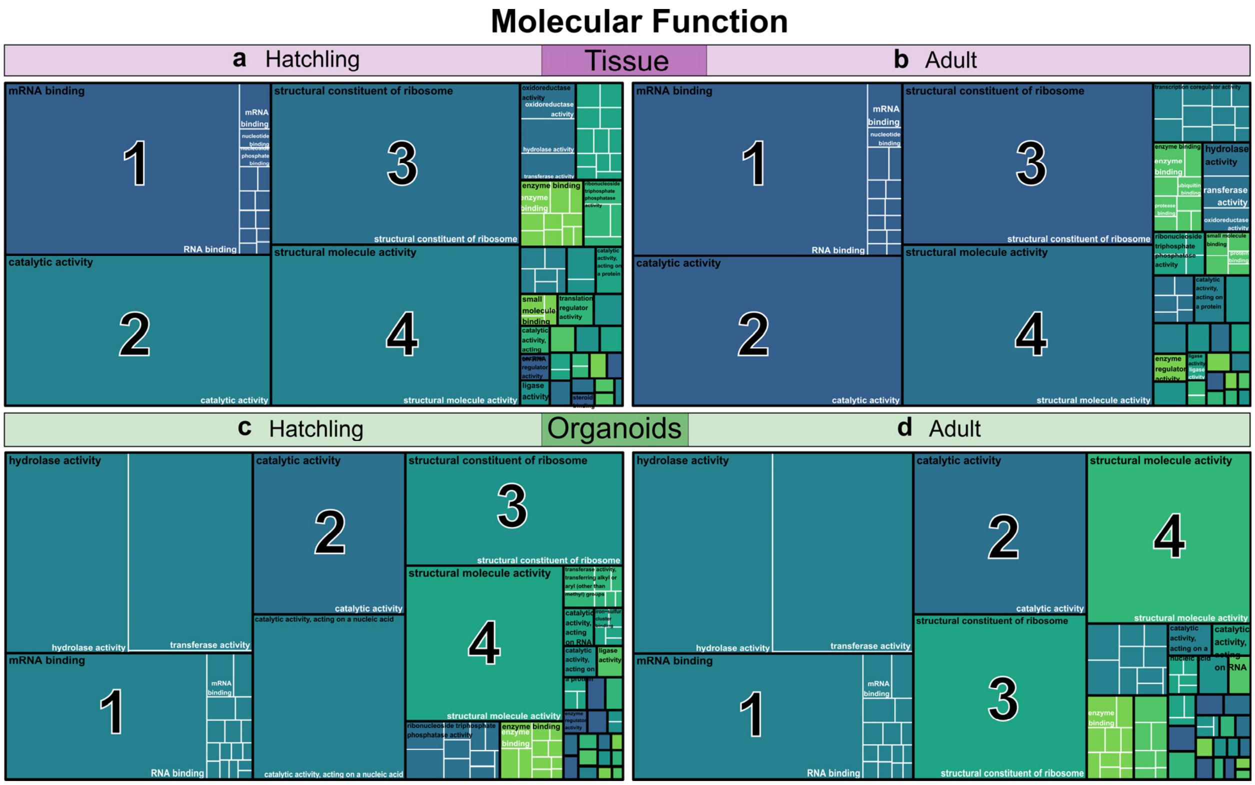

Molecular Functions of turtle liver tissue and organoids.

Supplementary Fig. 6: The Molecular Functions identified when analyzing the transcriptomic expression patterns of liver tissue and organoids. Enrichment analysis of **(a)** hatchling tissue (*n*=3), **(b)** adult tissue (*n*=3) samples of liver in addition to liver cultures of **(c)** hatchling organoid (*n*=3), **(d)** adult organoid (*n*=3) of *C. picta* liver samples. Top supercluster terms: (1) mRNA binding, (2) catalytic activity, (3) structural constituent of ribosome, (4) structural molecule activity.

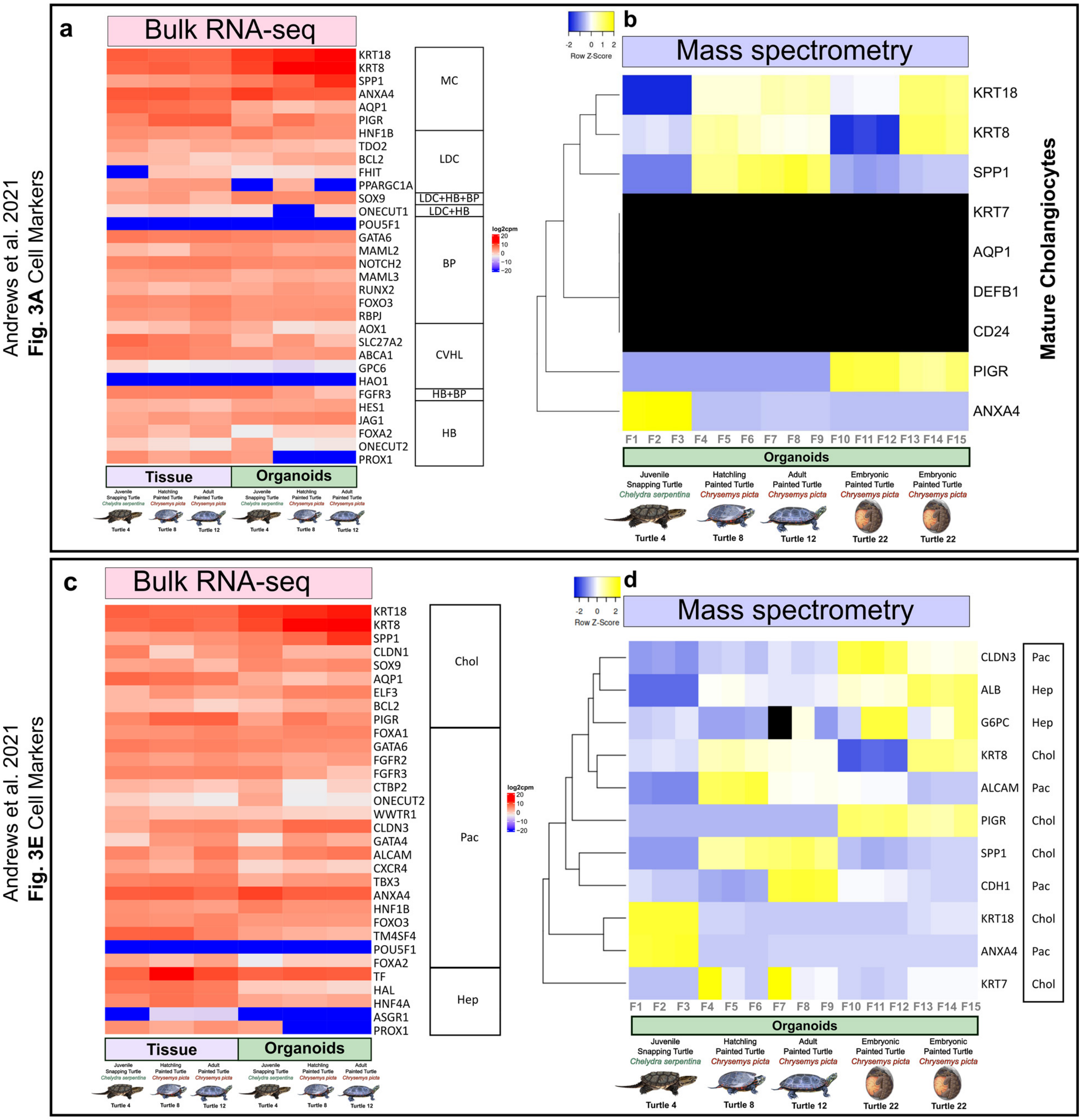

RNA and proteomic expression of know liver markers.

Supplementary Fig. 7: **(a,c)** Transcription (mRNA) and **(b,d)** proteomic expression of *C. serpentina* and *C. picta* for known liver markers[1]. **(a,c)** For RNA heatmaps, gene counts from the assembly including *C. picta* and *C. serpentina* were converted to counts per million with libraries normalized via TMM method. A log2 transformation was applied to the counts to correct for heteroskedasticity. These were then used as input for the heatmaps, with highly expressed (red) and lowly expressed (blue) being displayed. The corresponding cell marker pertaining to each gene are listed: LDC = less-differentiated cholangiocyte, MC = mature cholangiocytes, BP = bipotent progenitors, CVHL = central venous hepatocyte-like, and HB = hepatocyte-biased. **(b)** Only cluster 4 (Mature Cholangiocytes) had matches, many of these may not be translated or were below the detection limit of the mass spectrometry analysis (black boxes = lack of expression). **(c)** The corresponding cell marker pertaining to each gene are listed: Chol = cholangiocytes, Pac = progenitor-associated cells, and Hep = hepatocyte.

Supplementary References

1. Andrews, T. S. *et al.* Single-Cell, Single-Nucleus, and Spatial RNA Sequencing of the Human Liver Identifies Cholangiocyte and Mesenchymal Heterogeneity. *Hepatol Commun* **6**, 2022 (2021).
