# Supplementary material for: Establishment and characterization of turtle liver organoids provides a potential model to decode their unique adaptations
Source: Commun Biol. 2024 Feb 22;7:218. doi: 10.1038/s42003-024-05818-1 (PMC10883927; doi:10.1038/s42003-024-05818-1)
Supplement: Supplementary file 2 — Description of Additional Supplementary Files [file 42003_2024_5818_MOESM2_ESM.docx]

Description of Additional Supplementary Files

**File name:** Supplementary Data 1 (42003_2024_5818_MOESM4_ESM.xlsx)

**Description:** Sample information for attempted organoid cultures including ID, species, age, and sex. Passage number and outcomes for all attempted turtle organoid cultures. Details on which samples were harvested for characterization and cryopreserved are listed.

**File name:** Supplementary Data 2 (42003_2024_5818_MOESM5_ESM.xlsx)

**Description:** Stranded RNA-seq reads mapped per sample for both tissue and organoids.

**File name:** Supplementary Data 3 (42003_2024_5818_MOESM6_ESM.xlsx)

**Description:** Differential expression of genes of interest discussed in the text.

**File name:** Supplementary Data 4 (42003_2024_5818_MOESM7_ESM.xlsx)

**Description:** Raw values obtained from the single-nuclei RNA-seq analysis of embryonic painted turtle (*Chrysemys picta*) liver organoids used to create Figures 4c and 4d.

**File name:** Supplementary Data 5 (42003_2024_5818_MOESM8_ESM.xlsx)

**Description:** Media composition for CMGF+ used in the optimized cultures as well as concentrations, manufacturers, and additional growth factors are listed.
