# Supplementary material for: Establishment and characterization of turtle liver organoids provides a potential model to decode their unique adaptations
Source: Commun Biol. 2024 Feb 22;7:218. doi: 10.1038/s42003-024-05818-1 (PMC10883927; doi:10.1038/s42003-024-05818-1)
Supplement: Supplementary file 8 — Reporting Summary [file 42003_2024_5818_MOESM8_ESM.pdf]

nature portfolio

Corresponding author(s): Christopher Zdyrski and Nicole Valenzuela

Last updated by author(s): Dec 10, 2023

Reporting Summary

Nature Portfolio wishes to improve the reproducibility of the work that we publish. This form provides structure for consistency and transparency in reporting. For further information on Nature Portfolio policies, see our Editorial Policies and the Editorial Policy Checklist.

Statistics

For all statistical analyses, confirm that the following items are present in the figure legend, table legend, main text, or Methods section.

n/a	Confirmed	
☐	☒	The exact sample size (*n*) for each experimental group/condition, given as a discrete number and unit of measurement
☒	☐	A statement on whether measurements were taken from distinct samples or whether the same sample was measured repeatedly
☐	☒	The statistical test(s) used AND whether they are one- or two-sided<br>*Only common tests should be described solely by name; describe more complex techniques in the Methods section.*
☒	☐	A description of all covariates tested
☐	☒	A description of any assumptions or corrections, such as tests of normality and adjustment for multiple comparisons
☒	☐	A full description of the statistical parameters including central tendency (e.g. means) or other basic estimates (e.g. regression coefficient) AND variation (e.g. standard deviation) or associated estimates of uncertainty (e.g. confidence intervals)
☐	☒	For null hypothesis testing, the test statistic (e.g. *F*, *t*, *r*) with confidence intervals, effect sizes, degrees of freedom and *P* value noted<br>*Give P values as exact values whenever suitable.*
☒	☐	For Bayesian analysis, information on the choice of priors and Markov chain Monte Carlo settings
☒	☐	For hierarchical and complex designs, identification of the appropriate level for tests and full reporting of outcomes
☒	☐	Estimates of effect sizes (e.g. Cohen's *d*, Pearson's *r*), indicating how they were calculated

Our web collection on statistics for biologists contains articles on many of the points above.

Software and code

Policy information about availability of computer code

Data collection	Custom script are uploaded to GitHub at https://github.com/ValenzuelaLab/ZdyrskiEtAl2023_CommBiol_TurtleLiverOrganoids and DOI: 10.5281/zenodo.10359908
Data analysis	Reads were trimmed with trimmomatic (version 0.39) (Bolger et al., 2014) to remove low-quality bases and adapter contamination. Reads were checked post-trimming with FASTQC (v 0.11.7) (S. Andrews, 2010) to confirm quality. Following trimming, reads were mapped to the C. picta RefSeq genome (Chrysemys_picta_BioNano-3.0.4) (Lee et al., 2020) using GSNAP (version 2021-03-08) (Wu & Nacu, 2010; Wu & Watanabe, 2005). Read representation was calculated using samtools (version 1.10) (Li et al., 2009). Following mapping, individual library BAM files were genome-guided assembled with StringTie (version 1.3.4a) (Pertea et al., 2015) and then merged into a single assembly using the --merge function. Following merging, transcript abundances were calculated for each library and counts were extracted using the prepDE.py script.<br><br>In parallel, ERCC reads were mapped to the ERCC reference following the same assembly pipeline as for the sample reads, except that discovery of novel transcripts was not permitted during assembly. Counts for the ERCC transcripts were appended to the gene count matrix for C. picta. Differential expression of gene models was calculated with DESeq2 (version 1.24.0) (Love et al., 2014) for C. picta in R (version 4.0.2) (R Core Team, 2018), testing for the effect of age, sample type (adult organoid [n=3], hatchling organoid [n=3], adult tissue [n=3], hatchling tissue [n=3]), and their interaction via a full factorial generalized linear model (Y ~ Age * Sample Type). Estimation of size factors was based on ERCC spike-in transcripts for normalization of the data. As many genes showed an interaction effect, the full factorial model was retained. Differentially expressed genes were filtered based on a baseMean (mean of the counts for all samples that have been normalized for sequencing depth) of > 50 and a P-adjusted value < 0.05. Multiple comparisons were corrected with the FDR/Benjamini-Hochberg method.<br><br>Blastx (blast-plus version 2.7.1) (Camacho et al., 2009) against the Uniprot database (accessed May 24, 2022) (Consortium, 2019) was used to

further annotate transcripts that were not annotated during the initial genome-guided assembly, although some transcripts remained unannotated after this blastx. Transcript sequences were extracted from the transcriptome using gffread (v0.12.7) (Pertea & Pertea, 2020). These transcripts were then translated into peptide sequences using TransDecoder (version 5.5.0; https://github.com/TransDecoder/TransDecoder). These sequences were then searched against the PANTHERDB (v17.0) (Mi et al., 2019; Thomas et al., 2022) hidden Markov models to obtain compatible sequence identifiers for enrichment analysis. Stringtie transcript counts were converted to gene-level lengthScaledTPM using the tximport package (v1.18.1) (Soneson et al., 2015) in R. Genes were then mapped to their corresponding PANTHER IDs via transcript isoforms. In the case where multiple isoforms were present, the isoform with the best supported PANTHER ID was prioritized. Unannotated transcripts were filtered out of the analysis, as this was required by the program.
PANTHER IDs and corresponding expression values were submitted to pantherdb.org for statistical enrichment analysis (Released 2022-10-17) which uses a Mann-Whitney U test to calculate enrichment of GO terms. Enrichment analysis was performed for each library and was searched against the following databases (v17.0 Released 2022-02-22) (Ashburner et al., 2000; Carbon et al., 2021): Pathways, GO-Slim Molecular Function, GO-Slim Biological Process, GO-Slim Cellular Component, and Protein Class. GO terms were filtered for terms that were over-enriched (as opposed to under-enriched). Following filtering, terms were filtered for those present in all three biological replicates. These over-enriched and replicated terms were input into REVIGO (Supek et al., 2011) for visualization. REVIGO reduces redundancy in lists of GO terms by considering semantic similarity and identifies terms that are most representative of clusters of related terms. It then allows the user to generate graphical representations of these relationships to aid interpretation of the enrichment results. REVIGO was run with a cutoff of 0.5 and terms were provided with FDR values. Obsolete terms were removed, and the dataset was compared to the Whole Uniprot Database and used the SimRel semantic similarity measure. Analyses were run on 2022/12/09 and the databases used for reference were go.obo (2022-11-03) and goa_uniprot_gcrp.gaf.gz (2022-09-16). Treemap (Tennekes & Ellis, 2017) was used to visualize the resulting clusters. The REVIGO-provided Rscript was downloaded and used to generate plots for interpretation.

Single nuclei RNA libraries were generated using the Chromium Single Cell 3' kit (10X Genomics, CA, USA). Loading onto the Chromium Controller was performed to target capture of ~10,000 GEMs per sample for downstream analysis and processed through the Chromium Controller. Quality of the sequencing libraries were evaluated on the Agilent TapeStation, then quantified using a Qubit 2.0 Fluorometer (Invitrogen, Carlsbad, CA). Prior to loading onto an Illumina sequencing platform, pooled libraries were quantified using qPCR (Applied Biosystems, Carlsbad, CA, USA). The samples were sequenced at a configuration compatible with the recommended guidelines outlined by 10X Genomics. Raw sequence data (.bcl files) were converted into fastq files and de-multiplexed using the 10X Genomics' cellranger mkfastq command. Subsequent UMI and cell barcode de-convolution along with mapping to the reference genome Chrysemys_picta_bellii-3.0.3 (GCA_000241765.2) were performed using 10X Genomics Cell Ranger 6.0.1 (Zheng et al., 2017) software package to generate the final digital gene expression matrices and cloupe files.

snRNA-seq reads were mapped to the reference genome (Chrysemys_picta_BioNano-3.0.4) then the Seurat package (v. 4.0) (Hao et al., 2021) in R (v. 4.2) (R Core Team, 2018) was used. The percentage of reads mapping to mitochondrial genes was used to minimize mitochondrial contamination typically seen in low quality or dying cells, and those with a high percentage were filtered out. Cells were filtered to retain those with gene counts between 200 and 6000 and having less than 40% mitochondrial contamination. Next, the data was normalized using the log Normalization method. Then features with high cell to cell variation were identified. The data was then scaled so that the mean expressions across the cells were 0 and variance across the cells was 1 prior to performing a principal component analysis. Clustering of samples utilized the KNN method, and using default parameters, clusters were identified.

For manuscripts utilizing custom algorithms or software that are central to the research but not yet described in published literature, software must be made available to editors and reviewers. We strongly encourage code deposition in a community repository (e.g. GitHub). See the Nature Portfolio guidelines for submitting code & software for further information.

Data

Policy information about availability of data

All manuscripts must include a data availability statement. This statement should provide the following information, where applicable:

- Accession codes, unique identifiers, or web links for publicly available datasets
- A description of any restrictions on data availability
- For clinical datasets or third party data, please ensure that the statement adheres to our policy

The stranded mRNA raw RNA-seq reads and snRNA-seq reads generated and analyzed in this study are available in the Sequence Read Archive (NCBI-SRA BioProject PRJNA931617). Bioinformatic scripts are available on GitHub (https://github.com/ValenzuelaLab/ZdyrskiEtAl2023_CommBiol_TurtleLiverOrganoids) and DOI: 10.5281/zenodo.10359908.

Human research participants

Policy information about studies involving human research participants and Sex and Gender in Research.

Reporting on sex and gender

Use the terms sex (biological attribute) and gender (shaped by social and cultural circumstances) carefully in order to avoid confusing both terms. Indicate if findings apply to only one sex or gender; describe whether sex and gender were considered in study design whether sex and/or gender was determined based on self-reporting or assigned and methods used. Provide in the source data disaggregated sex and gender data where this information has been collected, and consent has been obtained for sharing of individual-level data; provide overall numbers in this Reporting Summary. Please state if this information has not been collected. Report sex- and gender-based analyses where performed, justify reasons for lack of sex- and gender-based analysis.

Population characteristics

Describe the covariate-relevant population characteristics of the human research participants (e.g. age, genotypic information, past and current diagnosis and treatment categories). If you filled out the behavioural & social sciences study design questions and have nothing to add here, write "See above."

Recruitment	*Describe how participants were recruited. Outline any potential self-selection bias or other biases that may be present and how these are likely to impact results.*
Ethics oversight	*Identify the organization(s) that approved the study protocol.*

Note that full information on the approval of the study protocol must also be provided in the manuscript.

Field-specific reporting

Please select the one below that is the best fit for your research. If you are not sure, read the appropriate sections before making your selection.

☒ Life sciences ☐ Behavioural & social sciences ☐ Ecological, evolutionary & environmental sciences

For a reference copy of the document with all sections, see nature.com/documents/nr-reporting-summary-flat.pdf

Life sciences study design

All studies must disclose on these points even when the disclosure is negative.

Sample size	Three samples were used for each of the categories including hatchling organoid, hatchling tissue, adult organoid, and adult tissue when comparing stranded bulk mRNA RNA-seq data.
Data exclusions	No data were excluded from the analyses.
Replication	Reproducibility for successful thawing of organoids was tested for multiple samples frozen in long-term storage. Our supplementary tables display the successfully and unsuccessful thawing attempts.
Randomization	This is not relevant to our study as organoid culture and analysis of RNAseq data do not require randomization.
Blinding	Blinding was not relevant to our study as organoid culture and analysis of RNAseq data do not require blinding.

Reporting for specific materials, systems and methods

We require information from authors about some types of materials, experimental systems and methods used in many studies. Here, indicate whether each material, system or method listed is relevant to your study. If you are not sure if a list item applies to your research, read the appropriate section before selecting a response.

Materials & experimental systems

n/a	Involved in the study	
☐	☒	Antibodies
☐	☒	Eukaryotic cell lines
☒	☐	Palaeontology and archaeology
☐	☒	Animals and other organisms
☒	☐	Clinical data
☒	☐	Dual use research of concern

Methods

n/a	Involved in the study	
☒	☐	ChIP-seq
☒	☐	Flow cytometry
☒	☐	MRI-based neuroimaging

Antibodies

Antibodies used	For immunohistochemistry, slides were stained for proliferating cell nuclear antigen (PCNA) (DAKO; 0879) at a 1:400 dilution, and images were taken on an ECHO Revolution microscope (ECHO)
Validation	Control tissue slides were ran alongside the turtle samples with positive and negative controls present for all samples. Staining was then assessed by a pathologist.

Eukaryotic cell lines

Policy information about cell lines and Sex and Gender in Research

Cell line source(s)	Organoid cell lines were all derived from fresh tissue. The sex of all samples attempted include: Turtle 1 - Not determined, Turtle 2 - Male, Turtle 3 - Male, Turtle 4 - Male, Turtle 5 - Not determined, Turtle 6 - Not determined, Turtle 7 - Not determined, Turtle 8 - Female, Turtle 9 - Female, Turtle 10 - Not determined, Turtle 11 - Male, Turtle 12 - Male, Turtle 13 - Male, Turtle 14 - Male, Turtle 15 - Not determined, Turtle 22 - Not determined.

Authentication	None of the cell lines used were authenticated.
Mycoplasma contamination	The cell lines were not tested for mycoplasma contamination.
Commonly misidentified lines (See ICLAC register)	*Name any commonly misidentified cell lines used in the study and provide a rationale for their use.*

Animals and other research organisms

Policy information about studies involving animals; ARRIVE guidelines recommended for reporting animal research, and Sex and Gender in Research

Laboratory animals	Samples were obtained from animals collected and temporarily stored in the laboratory. The three species included Apalone spinifera, Chelydra serpentina, and Chrysemys picta. Sample specific ages are listed, ranging from embryonic samples, hatchlings, to adults.
Wild animals	Collected eggs were transferred from the field to the laboratory where they were incubated at relevant temperatures. Other individuals were captured in traps prior to being transferred to the laboratory. All animals used were euthanized for sample collection following ISU-IACUC approved protocols, after which they were then washed in iodine and hydrogen peroxide, prior to tissue harvesting.
Reporting on sex	Gonadal inspection was used to determine the sex of a subset of the samples. Those without clear distinguishing features (Apalone spinifera and embryonic samples) were listed as not determined. Transcriptomic comparisons were not made between those with known sex and those with an undetermined sex.
Field-collected samples	Animals were collected in Iowa under appropriate permits from the Iowa DNR (SC648 and SC595), and all procedures followed protocols approved by the Institutional Animal Care and Use Committee (IACUC) (IACUC-21-121) of Iowa State University as described below. Details of the donor animals, including sex, age, and the outcome of the organoid culture, can be found in Supplemental Table S1. Adult males and freshly laid C. picta eggs were collected from the wild. Hatchlings and juveniles were obtained from eggs incubated in the laboratory at 26°C (C. picta), a temperature that produces exclusively males in painted turtles, as this species displays temperature-dependent sex determination (TSD). The embryonic sample at developmental stage 22 (Yntema, 1968), was obtained from a C. picta egg incubated at 26°C. Eggs of C. serpentina (TSD) were incubated at 27.5°C, which produces a mixed sex ratio (Ewert et al., 1994), such that sex of snapping turtle juveniles was diagnosed by gonadal inspection. In contrast, A. spinifera displays a ZZ/ZW sex chromosome system of genotypic sex determination (GSD) (Badenhorst et al., 2013) and produces both sexes at 27.5°C (Bull & Vogt, 1979), the temperature used here to incubate their eggs. Thus, A. spinifera juveniles can be sexed by PCR amplification of sex-linked markers (Literman et al., 2017), a simpler method than by qPCR of rDNA repeats (Literman et al., 2014).<br>Live animals were housed indoors in water tubs, provided with UV A/B bulbs and a dry surface for basking, and kept at ~24°C until processing. Animals were fed Tetra ReptoMin sticks ad libitum. Animals were euthanized, then washed in iodine and hydrogen peroxide, and sex was diagnosed (C. serpentina) or confirmed (C. picta and A. spinifera) by gross gonadal morphology or presumed by the incubation temperature (C. picta embryo).
Ethics oversight	Ethical approval for this study was obtained by the Institutional Animal Care and Use Committee (IACUC) (IACUC-21-121) of Iowa State University.

Note that full information on the approval of the study protocol must also be provided in the manuscript.
